# Hyper α2,6‐Sialylation Promotes CD4^+^ T‐Cell Activation and Induces the Occurrence of Ulcerative Colitis

**DOI:** 10.1002/advs.202302607

**Published:** 2023-07-09

**Authors:** Qingjie Fan, Mechou Li, Weiwei Zhao, Kaixin Zhang, Ming Li, Wenzhe Li

**Affiliations:** ^1^ Guangdong Provincial Key Laboratory of Infectious Diseases and Molecular Immunopathology Shantou University Medical College Shantou Guangdong 515041 China; ^2^ College of Basic Medical Science Dalian Medical University 9‐Western Section, Lvshun South Road Dalian Liaoning 116044 China

**Keywords:** α2,6‐sialylation, lipid rafts, nuclear factor‐κ‐gene binding, ST6GAL1, T‐cell receptor, ulcerative colitis

## Abstract

α2,6‐sialylation, catalyzed by α2,6‐sialyltransferase (ST6GAL1), plays a pivotal role in immune responses. However, the role of ST6GAL1 in the pathogenesis of ulcerative colitis (UC) remains unknown. ST6GAL1 mRNA is highly expressed in UC tissues compared with the corresponding adjacent normal tissues, and α2,6‐sialylation is significantly increased in the colon tissues of patients with UC. The expression of ST6GAL1 and proinflammatory cytokines, such as interleukin (IL)‐2, IL‐6, IL‐17, and interferon‐gamma, is also increased. The number of CD4^+^ T cells increases in UC patients. St6gal1 gene knockout (*St6gal1^−/‐^*) rats are established by clustered regularly interspaced short palindromic repeats (CRISPR)‐associated gene knockout system. St6gal1 deficiency reduces the levels of pro‐inflammatory cytokines and alleviates colitis symptoms in UC model rats. Ablation of α2,6‐sialylation inhibits the transport of the TCR to lipid rafts and suppresses CD4^+^ T‐cell activation. The attenuation of TCR signaling downregulates the expression of NF‐κB in ST6GAL^1‐/‐^ CD4^+^ T‐cells. Moreover, NF‐κB could bind to the ST6GAL1 promoter to increase its transcription. Ablation of ST6GAL1 downregulates the expression of NF‐κB and reduces the production of proinflammatory cytokines to relieve UC pathogenesis, which is a potential novel target for the clinical treatment of UC.

## Graphical abstract

α2,6‐sialylation regulates the entry of T‐cell receptors (TCRs) into lipid rafts, and then affects TCR signaling strength. Ablation of ST6GAL1 attenuates the TCR signaling, downregulates the expression of NF‐κB and reduces the production of proinflammatory cytokines, and relieves ulcerative colitis (UC) pathogenesis.

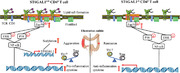

## 1 Introduction

Ulcerative colitis (UC) is a chronic, recurring mucosal inflammation of the colon characterized by flares that alternate with periods of remission.^[^ ^1^ ^]^ The severity of flares and their responses to treatment vary and are difficult to predict. The incidence of UC is increasing worldwide. Glycosylation is the most common post‐translational modification involved in the immune response and inflammation.^[^ ^2^ ^]^ Pham et al. reported that the expression of α1,2‐fucosyltransferase 2 (FUT2) in intestinal epithelial cells is important for maintaining intestinal homeostasis. *Fut2* deficiency promotes intestinal inflammation.^[^ ^2a^ ^]^ In addition, high levels of α1,6‐fucosyltransferase (FUT8) enhance T‐cell activation, inducing colitis.^[^ ^2b^ ^]^ Sialylation, which involves the terminal attachment of sialic acid to glycans with α2,3‐, α2,6‐, and α2,8‐linkages, is an important form of glycosylation.^[^ ^3^ ^]^ Each type of sialylation is catalyzed by corresponding sialyltransferases (STs), which transfer sialic acid from a cytidine‐5′‐monophosphate‐*N*‐acyl‐neuraminic acid (CMP‐Neu5Ac) donor to acceptor glycans.^[^ ^4^ ^]^ Several lines of evidence suggest that sialylation influences the immune response and intestinal inflammation.^[^ ^5^ ^]^ For instance, ST6GalNAc1 of mucin is important for maintaining intestinal homeostasis and colitis occurrence.^[^ ^5a^ ^]^ The levels of sialylated Lewis antigens increase in UC mice and promote epithelial cell proliferation and migration to repair damaged tissues.^[^ ^5b^ ^]^

**Figure 1:**
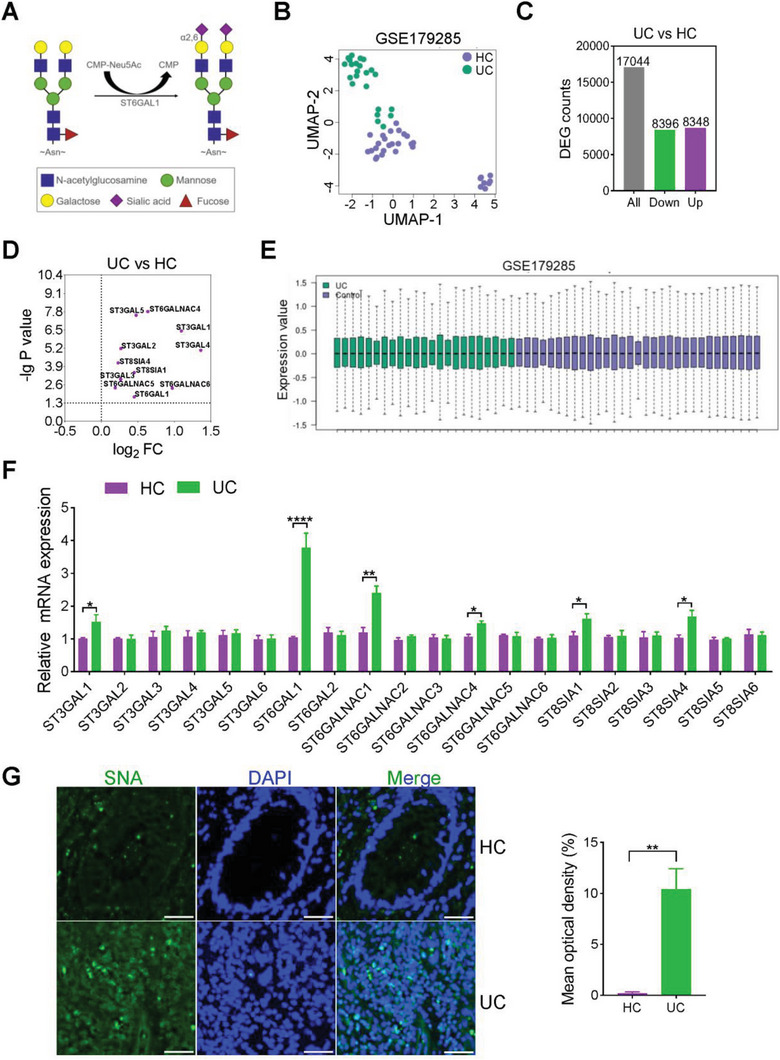
α2,6‐sialylation was significantly increased in UC colon tissues. A) Catalytic reaction of ST6GAL1: ST6GAL1 catalyzes the addition of sialic acid to Gal‐β1,4GlcNAc via α−2,6‐linkage. B) UMAP profiles of HCs (*n* = 31) and UCs (*n* = 23) from the GSE179285 database. C) Histogram of differentially‐expressed genes in UC compared with HCs in the GSE179285 database. D) Expression of sialyltransferase family genes in tissues with UC compared with HCs in the GSE179285 database. E) Box plots generated using the R‐boxplot algorithm for viewing the distribution of values for selected samples in the GSE179285 database. F) Real‐time PCR analysis of the 20 sialyltransferases: RNA was isolated from the HC and UC colon tissues (*n* = 25/group). G) Immunofluorescence analysis: The α2,6‐sialylation level in HC and UC colon tissue was determined by lectin blotting with SNA (*n* = 16/group). Scale bar, 100 µm. *p < 0.05, **p < 0.01, ***p < 0.001, ****p < 0.0001.

ST6GAL1 plays a crucial role in catalyzing the transfer of α2,6‐sialic acid to the Galβ1,4‐GlcNAc termini on the cell surface and secreted glycans (**Figure** **1**A).^[^ ^6^ ^]^ It is responsible for producing the Siaα2,6‐Galβ1,4‐GlcNAc terminus on *N*‐glycans and O‐glycans.^[^ ^7^ ^]^ ST6GAL1 has been implicated in various cellular processes, including cell apoptosis,^[^ ^8^ ^]^ cell stemness,^[^ ^9^ ^]^ cellular adhesion,^[^ ^10^ ^]^ B‐cell development,^[^ ^11^ ^]^ and inflammatory diseases.^[^ ^12^ ^]^ *St6gal1^−/‐^* mice exhibit dysregulated B cell development and reduced immunoglobulin production.^[^ ^11^ ^]^ *ST6GAL1* deficiency leads to increased lung inflammation with excessive eosinophilic infiltration.^[^ ^12^ ^]^ Ablation of *ST6GAL1* exacerbates acute neutrophilic inflammation in mice.^[^ ^13^ ^]^ Tumor necrosis factor (TNF) can inhibit *St6gal1* expression to decrease α2,6‐sialylation in synovial fibroblasts and promote the development of arthritis.^[^ ^14^ ^]^ However, the role of ST6GAL1 in UC remains unclear.

Dysregulation of CD4^+^ T‐cell function is associated with UC.^[^ ^2b^ ^]^ Our objective was to investigate the critical interplay between α2,6‐sialylation and CD4^+^ T‐cell activation and to elucidate the underlying mechanism of UC. Our findings highlight the significance of ST6GAL1 in CD4^+^ T‐cell activation and its involvement in the molecular mechanism underlying UC pathogenesis.

## 2 Results

### 2.1 α2,6‐Sialylation Is High in UC

Different classes of *N*‐glycans are catalyzed by several glycosidases and glycosyltransferases (Figure S1, Supporting Information). The most important “capping” reactions involve the addition of sialic acid to the branches by STs. To explore the correlation between sialylation and UC occurrence, we analyzed the expression of ST family genes in patients with UC compared with healthy controls (HCs) using Gene Expression Omnibus (GEO) database bioinformatics analysis (Figure 1B). In the GSE179285 dataset, 17 044 genes were altered in patients with UC compared with HCs, including 8396 upregulated and 8348 downregulated genes (Figure 1C). The expression levels of ST6GAL1, ST3GAL1, ST3GAL2, ST3GAL3, ST3GAL4, ST3GAL5, ST6GALNAC4, ST6GALNAC5, ST6GALNAC6, ST8SIA1, and ST8SIA4 were significantly increased in patients with UC (Figure 1D). The data were normalized across samples using the GEO2R analysis software for analysis of variance (Figure 1E). Abnormal sialylation has been observed in patients with UC or colon cancer.^[^ ^15^ ^]^ We isolated RNA from the colon tissues of patients with UC and HCs to compare the mRNA levels of STs. Compared with HCs, the mRNA expression of *ST6GAL1* was selectively overexpressed in patients with UC (Figure 1F). ST6GAL1 catalyzes the transfer of sialic acid from a CMP‐Neu5Ac donor to the terminal residue of galactose, forming an α2,6‐linkage (α2,6‐sialylation) (Figure 1A). We examined the α2,6‐sialylation level in situ by lectin blotting with Sambucus nigra agglutinin (SNA), which specifically recognizes the α2,6‐sialic acid structure.^[^ ^16^ ^]^ α2,6‐sialylation was mainly increased in the colon tissues of patients with UC (Figure 1G).

### 2.2 Ablation of ST6GAL1 Relieves UC Symptoms in Rats

**Figure 2:**
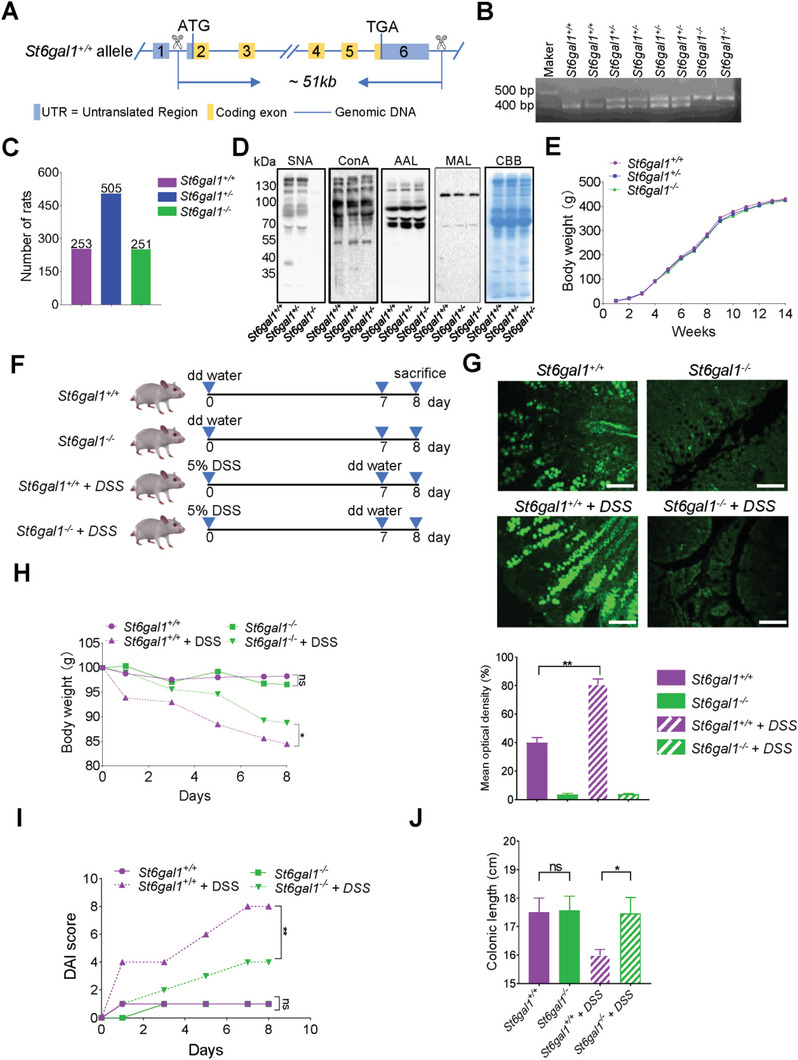
Ablation of ST6GAL1 relieves UC symptoms of UC rats. A) Overview of the *ST6GAL1* targeting strategy. B) *St6gal1^+/+^*, *St6gal1^+/−^*, and *St6gal1^−/−^* genotypes were confirmed by PCR. C) The ratio of *St6gal1^+/+^* (253), *St6gal1^+/‐^* (505), and *St6gal1* ^−/‐^ (251) offspring rats was ≈1:2:1. D) Lectin blot analysis: Glycosylation levels in the serum of *St6gal1^+/+^*, *St6gal1* ^+/−^, and *St6gal1* ^−/−^ rats were determined by lectin blotting with SNA, ConA, AAL, and MAL. Cell lysates were run on 10% SDS‐PAGE gels and immunoblotted. Coomassie brilliant blue (CBB) was used as a loading control. E) Body weight of *St6gal1^+/+^*, *St6gal1* ^+/‐^ and *St6gal1* ^−/‐^ rats (*n* = 15/group). F) Generating UC rats by administration of 5% DSS. G) Immunofluorescence analysis: The α2,6‐sialylation level in *St6gal1^+/+^*, *St6gal1* ^−/−^, *St6gal1^+/+^* UC, and *St6gal1^−/‐^* UC colon tissue was compared by lectin blotting with SNA (*n* = 15/group). H) Body weight: The body weights of *St6gal^+/+^* and *St6gal1^−/−^* rats were measured before and after UC induction (*n* = 15/group). I) DAI analysis: DAI scores in *St6gal^+/+^* and *St6gal1^−/−^* rats were determined before and after UC induction (*n* = 15/group). J) Colon lengths in *St6gal^+/+^* and *St6gal1^−/−^* male rats were measured before and after UC modeling (*n* = 15/group). Scale bar, 100 µm. *p < 0.05, **p < 0.01, ***p < 0.001.

Given that α2,6‐sialylation is significantly increased in patients with UC, we investigated how ST6GAL1‐mediated α2,6‐sialylation regulates UC pathogenesis. We engineered *St6gal1^+/−^* rats using the CRISPR‒Cas9 technique (**Figure** **2**A). Homozygous wild‐type (*St6gal1^+/+^*) and knockout (*St6gal1^−/^* ^−^) rats were obtained by crossing heterozygous *St6gal1^+/−^* rats (Figure 2B). When the *St6gal1* ^+/−^ rats were intercrossed, the ratio of *St6gal1^+/+^* (253), *St6gal1^+/‐^* (505), and *St6gal1* ^−/‐^ (251) rats was ≈1:2:1, corresponding to Mendelian inheritance (Figure 2C). ST6GAL1 products are α2,6‐sialylated *N*‐glycans, which were ubiquitously expressed in *St6gal1^+/+^* serum, as confirmed by SNA blotting, but were absent in *St6gal1^−/−^* serum (Figure 2D). No significant differences were observed in the lectin blot analysis of Aleuria aurantia lectin (AAL: specific for fucose), Maackia amurensis lectin (MAL: specific for Siaα2‐3 Gal), and concanavalin A lectin (ConA: specific for α‐Man/α‐Glc). Ablation of the *St6gal1* gene did not affect the body weight of the rats (Figure 2E).

To assess the role of ST6GAL1 in UC occurrence, we established a UC model using *St6gal1^+/+^* and *St6gal1^−/−^* rats administered with 5% dextran sulfate sodium (DSS) (Figure 2F). First, we detected the α2,6‐sialylation level in the rats using immunofluorescence. Α2,6‐Sialylation was increased in the colon tissue of UC rats (Figure 2G). Compared with *St6gal1^+/+^* rats, *St6gal1^−/−^* rats demonstrated significant weight loss in the UC model (Figure 2H). Moreover, *St6gal1^−/‐^* rats had mild stool irregularities and blood in the stool after UC modeling (Figure S2A, Supporting Information). The disease activity index (DAI) includes factors such as weight, stool shape, and blood in the stool and is often used to evaluate the severity of UC.^[^ ^17^ ^]^ The DAI scores of *St6gal1^‐/‐^* rats were lower than those of *St6gal1^+/+^* rats in the UC model (Figure 2I). In addition, the colon length was shorter in *St6gal1^+/+^* rats in the UC model, whereas *St6gal1^−/−^* rats revealed no change in colon length (Figure 2J and Figure S2B, Supporting Information), suggesting that ablation of ST6GAL1 relieved the symptoms of UC.

### 2.3 Expression of Proinflammatory Cytokines in CD4^+^ T‐Cells Is Upregulated in Patients with UC

**Figure 3:**
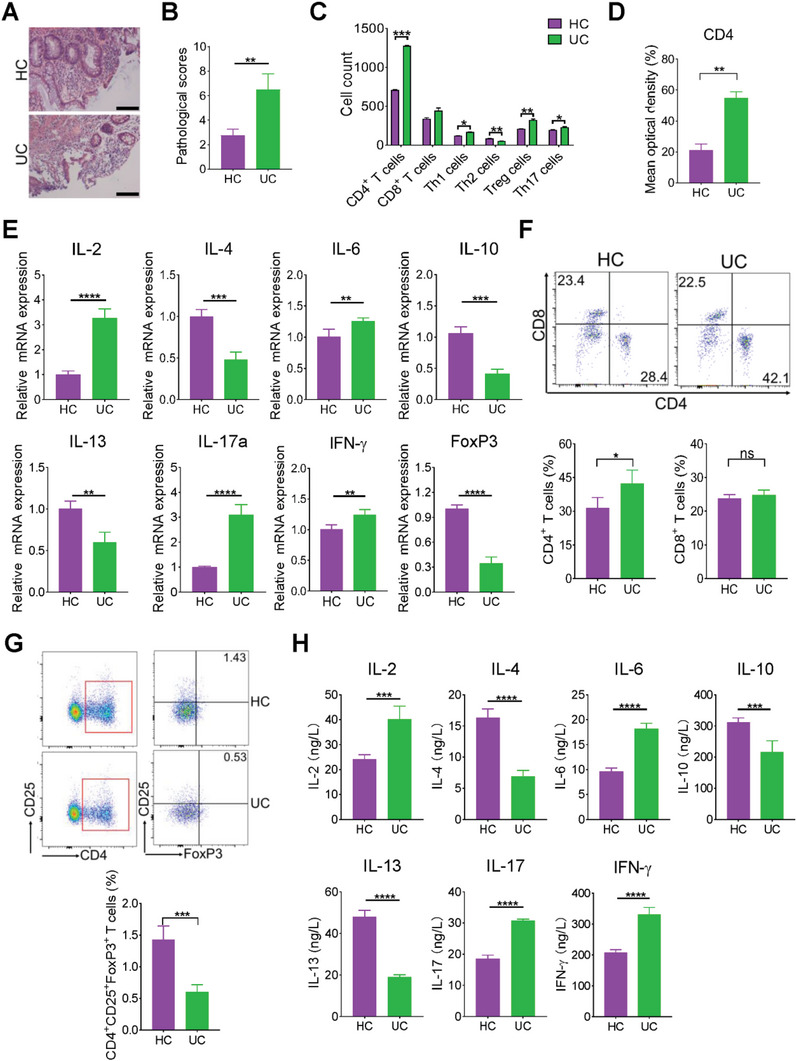
CD4^+^ T‐cell number and expression of proinflammatory cytokines by CD4^+^ T‐cells are upregulated in patients with UC. A) H&E staining of colon tissues with HCs and UC. B) Pathology score analysis of HCs and UC (*n* = 25/group). C) Cell counts: The number of CD4^+^ T, Th1, Th17, and Treg cells in the colon with HCs and UC (*n* = 3/group) was determined by single‐cell sequencing. D) Immunofluorescence analysis: CD4^+^ T‐cells in HC and UC colon tissue were analyzed (*n* = 20/group). E) Real‐time PCR: mRNA expression levels of cytokines in colons from HCs and UC were analyzed (*n* = 26/group). F) Flow cytometry: The percentage of CD4^+^ and CD8^+^ T‐cells in HCs and UC colon was determined (*n* = 25/group). G) The percentage of CD4^+^CD25^+^FoxP3^+^ T‐cells in the peripheral blood of HCs and UC was measured by flow cytometry (*n* = 20/group). H) ELISA analysis: Cytokine levels in the serum from HCs and UC were analyzed (*n* = 38/group). Scale bar, 200 µm. *p < 0.05, **p < 0.01, ***p  < 0.001.

Hematoxylin‐eosin staining (HE) staining showed severe colon damage in patients with UC (**Figure** **3**A) and increased pathology scores (Figure 3B). Moreover, we isolated intestinal epithelial cells from *St6gal1^+/+^* and *St6gal1^−/−^* rats, stimulated the intestinal epithelial cells with lipopolysaccharide (LPS) in vitro, and characterized the mRNA levels of ST6GAL1. α2,6‐sialylation increased in epithelial cells following LPS stimulation (Figure S2C, Supporting Information). Given that immune imbalance is one of the important reasons for UC pathogenesis,^[^ ^18^ ^]^ we analyzed the number of immune cells in patients with UC using single‐cell sequencing technology and the huARdb (https://huarc.net/database).^[^ ^19^ ^]^ The numbers of CD4^+^ T, Th1, Th17, and Treg cells increased in patients with UC, whereas the number of Th2 cells decreased (Figure 3C and Figure S2D, Supporting Information). Moreover, the number of CD4^+^ T cells drastically increased in the colon tissue (Figure 3D and Figure S3A, Supporting Information). Gene ontology (GO) enrichment analysis also suggested that CD4^+^ T cells and inflammatory responses were enriched in patients with UC (Figure S3B, Supporting Information). The levels of proinflammatory cytokines, such as interleukin (IL)−2, IL‐6, IL‐17a, and IFN‐γ, were significantly increased, while the levels of the anti‐inflammatory cytokines IL‐4, IL‐10, and IL‐13 were decreased in UC tissue (Figure 3E).

Next, we determined the number of immune cells in the peripheral blood of patients with UC. The number of CD4^+^ T cells significantly increased in patients with UC; however, no changes were observed in the number of CD8^+^ T cells (Figure 3F). The number of CD4^+^CD25^+^FoxP3^+^ (Treg) cells (Figure 3G) was decreased. The levels of pro‐inflammatory cytokines such as IL‐2, IL‐6, and IL‐17 were markedly increased, whereas the levels of the anti‐inflammatory cytokines IL‐4, IL‐10, and IL‐13 were decreased (Figure 3H). Overall, patients with UC had increased levels of proinflammatory cytokines, accompanied by the downregulation of anti‐inflammatory cytokines produced by CD4^+^ T cells.

### 2.4 Ablation of ST6GAL1 Gene Reduces the Production of Proinflammatory Cytokines by CD4^+^ T‐Cells in UC Rats

**Figure 4:**
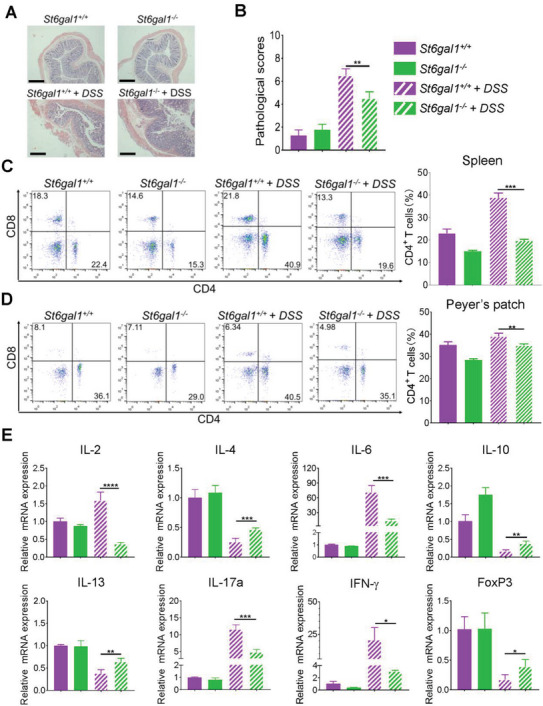
Ablation of ST6GAL1 gene reduced the production of proinflammatory cytokines by CD4^+^ T‐cells in UC model rats. A) H&E staining. The colon tissues were collected from *St6gal^+/+^* and *St6gal1^−/−^* rats before and after UC modeling. B) Pathology scores of *St6gal^+/+^* and *St6gal1^−/−^* male rats were analyzed before and after UC modeling (*n* = 15/group). C) Flow cytometry of splenic cells: The percentage of CD4^+^ and CD8^+^ T‐cells in the spleen of *St6gal^+/+^* and *St6gal1^−/−^* male rats before and after UC modeling were analyzed (*n* = 10/group). D) Flow cytometry of lymphocytes in the Peyer's patches: The percentage of CD4^+^ and CD8^+^ T‐cells of *St6gal^+/+^* and *St6gal1^−/−^* male rats before and after UC modeling were analyzed (*n* = 10/group). E) qPCR analysis: mRNA expression levels of cytokine in the colon from *St6gal^+/+^* and *St6gal1^−/−^* male rats were measured before and after UC modeling (*n* = 10/group). Scale bar, 200 µm. *p < 0.05, **p < 0.01, ***p < 0.001, ****p < 0.0001.

*St6gal1^−/−^* UC rats exhibited alleviated UC symptoms, and patients with UC revealed a significant increase in CD4^+^ T cells and excessive proinflammatory cytokines. This suggests that *ST6GAL1* affects the number of CD4^+^ T cells and cytokine levels. Therefore, we examined the number of CD4^+^ T cells and proinflammatory cytokine levels in *St6gal1^+/+^* and *St6gal1^−/−^* UC rats. *St6gal1^−/−^* UC rats revealed lower pathological scores than *St6gal1^+/+^* UC rats (**Figure** **4**A,B). Compared with *St6gal1^+/+^* UC rats, the number of CD4^+^ T cells decreased in both the spleen (Figure 4C) and Peyer's patches (Figure 4D) of *St6gal1^−/−^* UC rats. Moreover, CD4^+^ T‐cells derived from *St6gal1^+/+^* UC rats promoted the occurrence of UC compared with *St6gal1^−/−^* CD4^+^ T‐cells (Figure S4A–C, Supporting Information).

Several studies have reported that the balance between Treg and Th17 cells is important for maintaining intestinal homeostasis. A decrease in Treg cells or an increase in Th17 cells promotes intestinal inflammation.^[^ ^20^ ^]^ The numbers of Treg cells in the spleen (Figure S5A, Supporting Information) and Peyer's patches (Figure S5B, Supporting Information) increased in *St6gal1^−/−^* UC rats, whereas the number of Th17 cells decreased (Figure S5C, Supporting Information). The number of Treg cells also increased in *St6gal1^−/−^* CD4^+^ T cells, whereas the number of Th17 cells decreased (Figure S6A–C, Supporting Information). Moreover, *St6gal1^−/−^* UC rats exhibited low levels of proinflammatory cytokines IL‐2, IL‐6, IL‐17a, and IFN‐γ and high levels of the anti‐inflammatory cytokines IL‐4, IL‐10, and IL‐13 (Figure 4E). These results indicate that *ST6GAL1* ablation can induce the polarization of CD4^+^ T cells toward Tregs and inhibit their polarization toward Th17 cells.

### 2.5 Ablation of the ST6GAL1 Gene Inhibits the Proliferation and Activation of CD4^+^ T‐Cells

**Figure 5:**
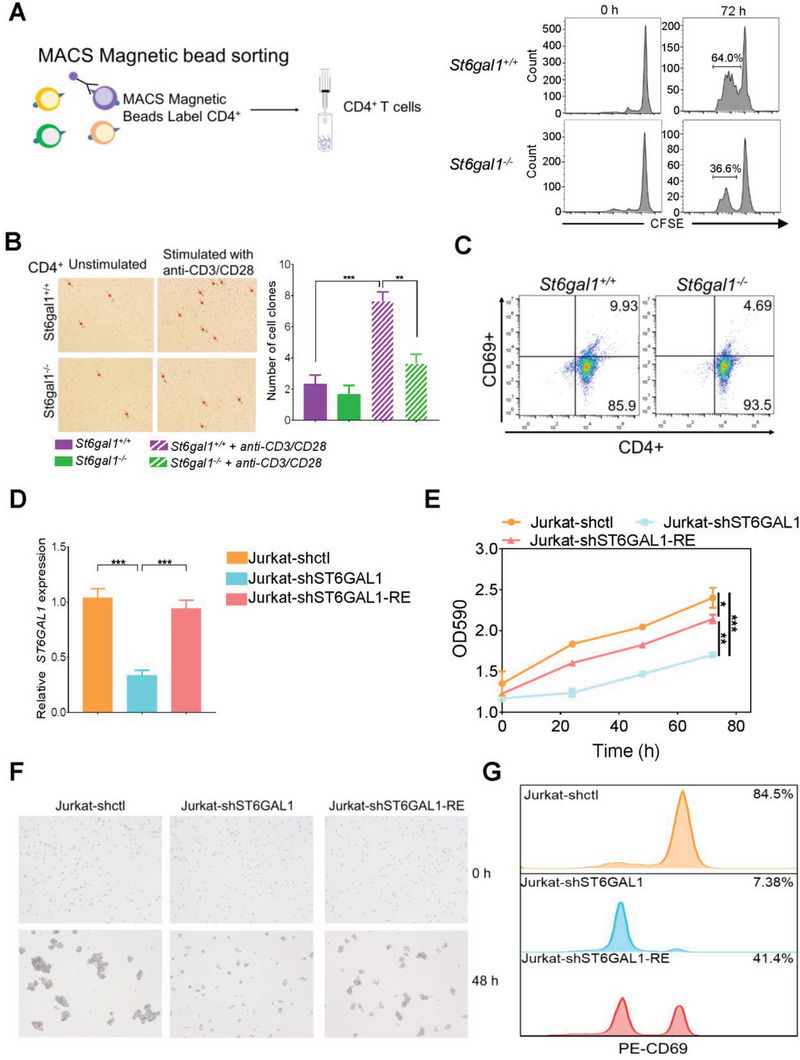
Ablation of the *ST6GAL1* gene inhibits the proliferation and activation of CD4^+^ T‐cells in vitro. A) CFSE assay: Cell proliferation of CD4^+^ T‐cells from *St6gal1^+/+^* and *St6gal1^−/−^* spleen were measured after incubation with anti‐CD3/CD28 Abs for 72 h (*n* = 5/group). B) Colony formation assay: Numbers of CD4^+^ T‐cell colonies from *St6gal1^+/+^* and *St6gal1^−/−^* spleens were counted after incubation with anti‐CD3/CD28 mAbs for 48 h (*n* = 5/group). C) Flow cytometry: Percentages of CD4^+^CD69^+^ T‐cells from the spleens of *St6gal^+/+^* and *St6gal1^−/−^* rats were determined after incubation with anti‐CD3/CD28 for 48 h (*n* = 5/group). D) Real‐time PCR analysis: ST6GAL1 mRNA expression levels in Jurkat‐shctl, Jurkat‐shST6GAL1, and Jurkat‐shST6GAL1‐RE cells were measured. E) CCK‐8 assay: Cell proliferation of Jurkat‐shctl, Jurkat‐shST6GAL1, and Jurkat‐shST6GAL1‐RE cells was measured after incubation with anti‐CD3 and anti‐CD28 Abs for 72 h (*n* = 5/group). F) Colony formation assay: Colony numbers of Jurkat‐shctl, Jurkat‐shST6GAL1, and Jurkat‐shST6GAL1‐RE cells were measured after incubation with anti‐CD3 and anti‐CD28 Abs for 48 h (*n* = 5/group). G) Flow cytometry: Percentages of CD69^+^ cells from Jurkat‐shctl, Jurkat‐shST6GAL1, and Jurkat‐shST6GAL1‐RE cells were determined after incubation with anti‐CD3 and CD28 Abs for 48 h (*n* = 5/group). *p < 0.05, **p < 0.01, ***p < 0.001.

The hyperactivation of CD4^+^ T cells is associated with UC pathogenesis.^[^ ^20^ ^]^ To investigate the role of ST6GAL1 in CD4^+^ T cell activation, we isolated CD4^+^ T cells from the spleens of *St6gal1^+/+^* and *St6gal1^−/−^* rats using magnetic bead sorting. In the carboxyfluorescein diacetate succinimidyl ester assay, ablation of *St6gal1* suppressed CD4^+^ T‐cells after stimulation with anti‐CD3 and CD28 Abs (**Figure** **5**A). T cells often aggregate into clusters after activation.^[^ ^21^ ^]^ Compared with *St6gal1^+/+^* CD4^+^ T cells, *St6gal1^−/‐^* CD4^+^ T cell aggregation was reduced following activation (Figure 5B). Flow cytometry also revealed a low number of CD4^+^CD69^+^ cells (activated CD4^+^ T cells) in *St6gal1^−/^* ^‐^ rats (Figure 5C).

To further clarify the mechanism by which ST6GAL1 regulates CD4^+^ T cell activation, we established *ST6GAL1* gene knockdown Jurkat cells (Jurkat‐shST6GAL1 cells), and *ST6GAL1* restored Jurkat‐shST6GAL1‐RE cells. ST6GAL1 mRNA expression was reduced in Jurkat‐shST6GAL1 cells and was restored by the reintroduction of the ST6GAL1 gene into Jurkat‐shST6GAL1 cells (Figure 5D). SNA lectin blot analysis confirmed ST6GAL1 gene expression levels. α2,6‐sialylation was barely detectable in Jurkat‐shST6GAL1 cells but was restored in Jurkat‐shST6GAL1‐RE cells (Figure S5D, Supporting Information). The loss of *ST6GAL1* resulted in the suppressed proliferation of Jurkat cells, which was restored by the reintroduction of ST6GAL1 (Figure 5E). Cell clustering was inhibited in Jurkat‐shST6GAL1 cells but recovered in Jurkat‐shST6GAL1‐RE cells (Figure 5F). The number of CD69^+^ cells was suppressed in Jurkat‐shST6GAL1 cells and recovered by the reintroduction of the ST6GAL1 (Figure 5G).

### 2.6 Ablation of ST6GAL1 Gene Suppresses T‐Cell Receptor (TCR) Translocation to Lipid Rafts and Attenuates TCR Signaling in CD4^+^ T‐Cells

**Figure 6:**
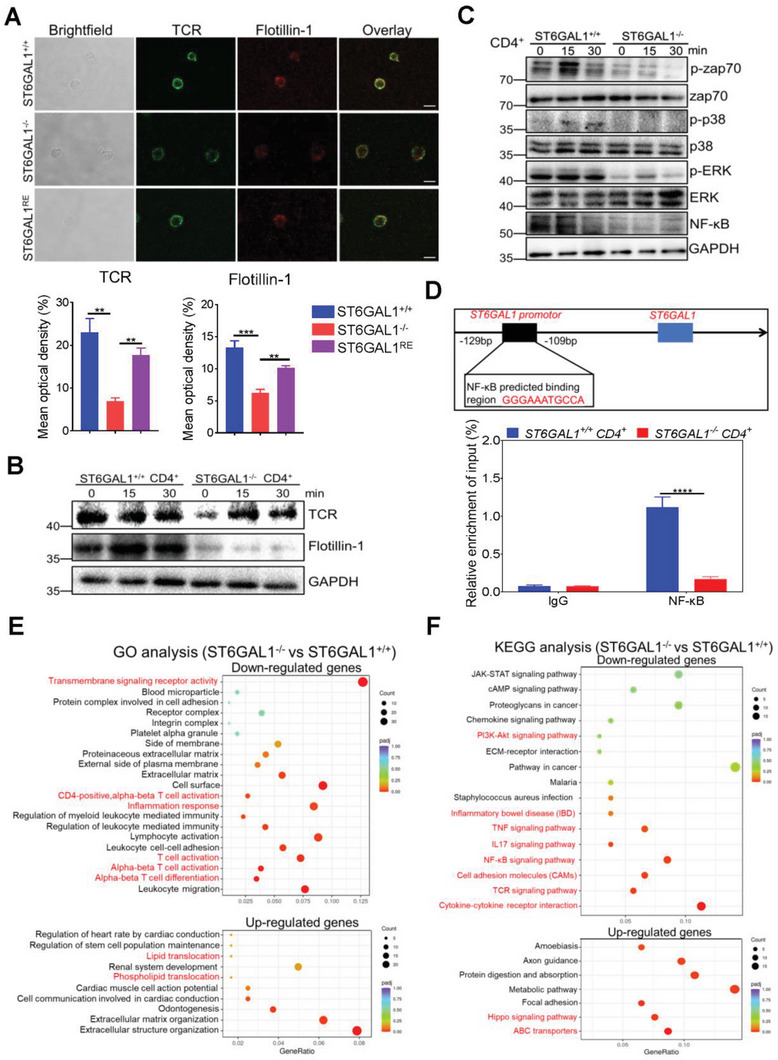
Ablation of the *ST6GAL1* gene suppresses TCR translocation to lipid rafts and attenuates TCR signaling in CD4^+^ T‐cells. A) Immunofluorescence staining: CD4^+^ T‐cells from *St6gal1^+/+^ and St6gal1^−/−^* splenic cells and ST6GAL1‐restored cells (ST6GAL1^RE^) were probed with anti‐TCR (green) and Flotillin‐1 (red) after stimulation with anti‐CD3/CD28 Abs for 15 min. B) Western blot analysis: CD4^+^ T cells from *St6gal1^+/+^ and St6gal1^−/−^* rats were incubated with anti‐CD3/CD28 Abs for 15 and 30 min, and expression levels of TCR and Flotillin‐1 in the lipid raft fraction were analyzed. Cell lysates were run on 10% SDS‐PAGE gels and immunoblotted. GAPDH was used as a loading control. C) Signaling pathway via TCR: *St6gal1^+/+^ and St6gal1^−/‐^* CD4^+^ T‐cells were incubated with anti‐CD3/CD28, and the phosphorylations of Zap70, p38, ERK, and the expression of NF‐κB were detected. Cell lysates were run on a 10% SDS‐PAGE gel and immunoblotted. GAPDH was used as a loading control. D) ChIP‐qPCR: Binding of NF‐κB to the *ST6GAL1* gene promoter was analyzed (*n* = 3/group). E) Bubble plots of GO enrichment analysis: Differentially‐expressed genes between *St6gal1^−/‐^* CD4^+^ T‐cells and *St6gal1^+/+^* CD4^+^ T‐cells were compared (*n* = 3/group). F) Bubble plots of KEGG enrichment analysis: Differentially‐expressed genes between *St6gal1^−/‐^* CD4^+^ T‐cells and *St6gal1^+/+^* CD4^+^ T‐cells were compared (*n* = 3/group). Scale bar, 50 µm. ****p < 0.0001.

In response to T‐cell activation, TCRs become associated with lipid rafts, which act as platforms for signaling and trafficking.^[^ ^22^ ^]^ We first determined the *N*‐glycan profile of the TCRs of CD4^+^ T cells using mass spectrometry. Sialylated *N*‐glycans bearing mono‐, di‐, tri‐, or tetra‐galactoses were identified in the TCRs. The α2,6‐sialylation of *N*‐glycans on TCRs was eliminated in *St6gal1^−/‐^* CD4^+^ T‐cells (Figure S5, Supporting Information). Confocal microscopy was used to examine the distribution of TCRs on CD4^+^ T cell surfaces after anti‐CD3/CD28 stimulation. Flotillin‐1 is a marker of lipid rafts that has an important effect on their structure and function of lipid rafts.^[^ ^23^ ^]^ Ablation of *St6gal1* inhibited lipid raft formation, as evidenced by the low expression of Flotillin‐1 (**Figure** **6**A,B). The level of TCRs in lipid rafts was decreased in *St6gal1^−/‐^* CD4^+^ T cells compared with *St6gal1^+/+^* CD4^+^ T cells, and these effects were rescued by the reintroduction of *ST6GAL1* (Figure 6A).

We examined the TCR signaling pathway after stimulation with anti‐CD3/CD28. Ablation of the *ST6GAL1* gene inhibited the phosphorylation of Zap70, p38, and extracellular regulated protein kinases (ERK), which are downstream of the TCR (Figure 6C). The expression of NF‐κB (p65) was downregulated in *St6gal1^−/‐^* CD4^+^ T‐cells (Figure 6C). Moreover, the loss of *St6gal1* inhibited p‐p65 nuclear translocation in CD4^+^ T cells (Figure S5, Supporting Information). The activation of NF‐κB often induces the production of proinflammatory cytokines, which induces UC;^[^ ^24^ ^]^ therefore, we analyzed the interaction between NF‐κB and *ST6GAL1*. chromatin immunoprecipitation‐real‐time polymerase chain reaction (ChIP‒qPCR) revealed that NF‐κB could bind upstream of the *ST6GAL1* promoter at −129 to −109 base pairs (bp). Compared with *St6gal1^+/+^* CD4^+^ T‐cells, the interaction between NF‐κB and *ST6GAL1* was suppressed in *St6gal1^−/‐^* CD4^+^ T cells (Figure 6D).

To further examine the role of *St6gal1* during T cell activation, we analyzed gene expression in *St6gal1^+/+^* and *St6gal1^−/‐^* CD4^+^ T cells using transcriptomics assay. Compared with *St6gal1^+/+^* CD4^+^ T cells, *St6gal1^−/−^* CD4^+^ T cells revealed 555 downregulated and 551 upregulated genes (Figure S9A,B, Supporting Information). GO analysis revealed that deletion of *St6gal1* suppressed CD4^+^ T cell activation and inflammatory responses (Figure 6E). Kyoto Encyclopedia of Genes and Genomes (KEGG) analysis revealed that loss of *St6gal1* downregulated the expression of multiple signaling molecules, such as the TCR, NF‐κB, IL‐17, TNF, and PI3K‐AKT (Figure 6F). Conversely, tissue transcriptomic analysis revealed that the TNF, IL‐17, NF‐κB, and PI3K‐AKT pathways were enhanced in patients with UC (Figure S9C,D, Supporting Information). These results suggest that *St6gal1* affects the activation of CD4^+^ T cells by regulating the TCR, NF‐κB, and other signaling pathways.

Loss of *ST6GAL1* suppressed TCR expression and lipid raft formation in Jurkat‐shST6GAL1 cells in a manner that could be rescued by reintroduction of the *ST6GAL1* into Jurkat‐shST6GAL1 cells (Figure S10A, Supporting Information). The western blotting results were consistent with the immunofluorescence results (Figure S10B, Supporting Information). Loss of *ST6GAL1* inhibited phosphorylation of Zap70, p38, and ERK and downregulated expression of NF‐κB in Jurkat‐shST6GAL1 cells, and these changes were restored in Jurkat‐shST6GAL1‐RE cells (Figure S10C, Supporting Information). CHIP‐qPCR assays revealed that NF‐κB bound to the upstream region of the *ST6GAL1* promoter at positions −129 to −109 bp in the Jurkat cell line and CD4^+^ T cells (Figure S10D, Supporting Information). We performed transcriptomic analysis to analyze the effect of the ST6GAL1 gene on the gene expression profile of Jurkat cells. Compared with Jurkat‐shctl cells, the expression of 850 genes was upregulated, and 1590 genes were downregulated (Figure S11A,B, Supporting Information). GO analysis revealed that knockdown of the *ST6GAL1* gene reduced T cell activation, cell surface receptor signaling, inflammatory response, and other biological functions (Figure S11C, Supporting Information). KEGG analysis revealed that *ST6GAL1* gene silencing reduced TCR, TNF‐, IL‐17, Extracellular Matrix (ECM), and other signaling pathways (Figure S11D, Supporting Information).

### 2.7 ST6GAL1 Is Positively Correlated with NF‐κB in UC Pathogenesis

**Figure 7:**
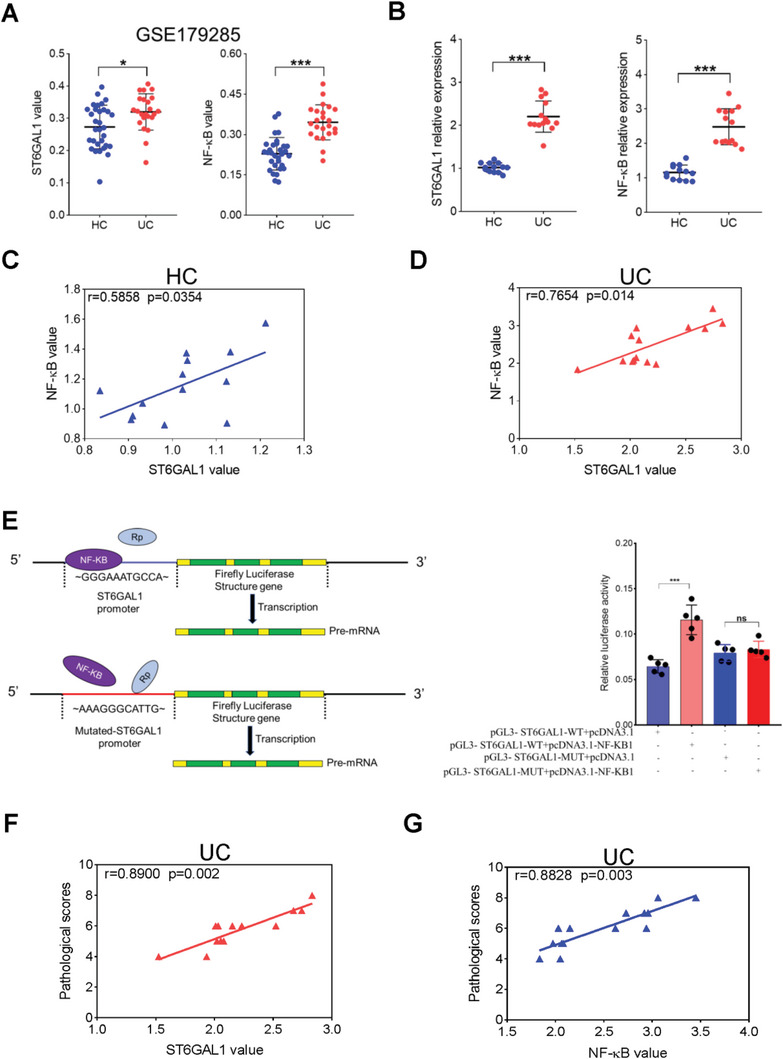
ST6GAL1 is positively correlated with NF‐κB in UC pathogenesis. A) ST6GAL1 and NF‐κB expression in the colonic mucosa of HCs (*n* = 31) and UCs (*n* = 23). Data from GSE179285 in the Gene Expression Omnibus database was retrieved. B) Real‐time PCR: mRNA expression of ST6GAL1 and NF‐κB in the colonic mucosa of HCs (*n* = 13) and UCs (*n* = 14). C,D) Intestinal biopsy samples were collected from HCs (*n* = 13) and patients with UC (*n* = 14). Correlation between intestinal ST6GAL1 and NF‐κB in C) HCs and D) patients with UC were analyzed. E) Dual‐luciferase reporter gene assay. The regulatory effect of NF‐κB on the promoter of the ST6GAL1 gene was determined. F,G) Spearman correlation analysis. The correlation between colonic F) ST6GAL1, G) NF‐κB, and colonic pathological score in patients with UC was determined. *p < 0.05, **p < 0.01, ***p < 0.001.

To investigate the correlation between *ST6GAL1* and *NF‐κB* in UC pathogenesis, we retrieved data from GSE179285 in the GEO database. The expression levels of *ST6GAL1* and *NF‐κB* were increased in the colons of patients with UC compared with HCs (**Figure** **7**A). Spearman correlation analysis revealed that *ST6GAL1* and *NF‐κB* were positively correlated in patients with UC and HCs (Figure S12A,B, Supporting Information). To further verify the correlation between *NF‐κB* and *ST6GAL1*, we measured the mRNA levels of *NF‐κB* and *ST6GAL1* in the colon tissues of 13 healthy volunteers and 14 patients with UC using RT‐qPCR. The expression of *NF‐κB* and *ST6GAL1* was significantly increased in the UC group (Figure 7B), and their expression was positively correlated (Figure 7C,D). We further verified the interaction between *ST6GAL1* and *NF‐κB* using a dual‐luciferase reporter assay. *NF‐κB* bound to the promoter region of the *ST6GAL1* gene and initiated transcription, resulting in a significant increase in luciferase activity (Figure 7E) and indicating that *NF‐κB* is strongly positively correlated with *ST6GAL1* gene expression. Moreover, the expression of *NF‐κB* and *ST6GAL1* was positively correlated with UC severity(Figure 7F,G), suggesting that the development of inhibitors of *NF‐κB* and *ST6GAL1* may be useful for treating UC.

## 3 Discussion

Inflammation is an important factor in UC pathogenesis.^[^ ^18^ ^]^ Intestinal inflammation is associated with cellular glycosylation.^[^ ^2^, ^5^, ^6^, ^7^ ^]^ We demonstrated that patients with UC exhibit high α2,6‐sialylation, which mediates the observed dysregulation of CD4^+^ T cell function in UC. Alterations in ST6GAL1 expression influence the balance of Th1/Th2 and Treg/Th17 cells by regulating the expression of pro‐ and anti‐inflammatory cytokines.

Glycosylation has evolved into a larger regulatory network that controls T‐cell activation.^[^ ^25^ ^]^ Differential effects of glycosylation on specific T‐cell lineages have been reported previously.^[^ ^2^, ^26^ ^]^ For instance, *N*‐acetylglucosaminyltransferase V (which catalyzes the synthesis of β1,6 GlcNAc branched *N*‐glycans) has been associated with the development of autoimmunity due to T‐cell hyperactivity.^[^ ^26a^ ^]^ Loss of ST3GAL1 (which catalyzes a2,3‐sialylation) increases the sensitivity of TCRs to their ligands in CD8^+^ T cells.^[^ ^26b^ ^]^ Fucosyltransferase 1 (FUT1, which catalyzes the addition of a1,2‐fucose to glycans)‐transgenic mice exhibited increased TCR signaling and apoptosis in thymocytes.^[^ ^26c^ ^]^ Core fucosylation catalyzed by FUT8 in T cells increases in patients with inflammatory bowel disease.^[^ ^2b^ ^]^ Previously, we discovered that loss of FUT8 impaired the interaction between CD4^+^ T and B cells.^[^ ^27^ ^]^ This evidence of T‐cell response disorders allowed us to address whether the dysregulation of α2,6‐sialylation contributes to T‐cell activity and the mechanism underlying UC. The TCR contains *N*‐glycans at Asn^70^, Asn^185^, and Asn^203^ in the α‐chain (Cα) and Asn^236^ in the β‐chain (Cβ).^[^ ^28^ ^]^ Herein, we determined the profile of *N*‐glycans on TCRs using mass spectroscopy and revealed that high α2,6‐sialylation of TCRs induced hyperactivation of CD4^+^ T cells during UC pathogenesis.

CD4^+^ T‐cells are usually determined by TCR entry into lipid rafts, which act as platforms for the signaling and trafficking of TCRs.^[^ ^29^ ^]^ In this study, we revealed that α2,6‐sialylation of TCR regulates the efficient coalescence of the lipid raft. The decreased Flotillin‐1 expression due to *ST6GAL1* loss could be recovered by reintroducing the *ST6GAL* gene. We propose the following mechanism for the reduction in lipid raft formation due to *ST6GAL1* disruption. First, because the domain of lipid rafts is hydrophobic^[^ ^30^ ^]^ and reduction in sialylation increases hydrophilicity, the loss of *ST6GAL1* suppresses TCR association with lipid rafts. Second, sialylation is most commonly α‐linked; therefore, it protrudes away from β‐linked ribbon‐like poly‐*N*‐acetyllactosamine branches;^[^ ^4^ ^]^ thus, α2,6‐sialylation could directly influence the interaction between molecules associated with lipid rafts. α2,6‐sialylation regulates TCR associations with lipid rafts at 15 min and signal transduction via TCR at 30 min, indicating that lipid raft formation on the CD4^+^ T cell membrane precedes signaling through TCR. The phosphorylation of ZAP70, ERK, p38, and NF‐κB (p65), which are downstream of the TCR, is attenuated by the loss of *ST6GAL1*, and the decreased signaling in *Jurkat‐shST6GAL1* cells is proportional to the decreased formation of lipid rafts. α2,6‐sialylation catalyzed by ST6GAL1 may regulate the entry of TCRs into lipid rafts and affect signaling strength via TCR in CD4^+^ T‐cell activation.

To further explore the possible mechanism of the dysregulation of ST6GAL1 in UC pathogenesis, we established *St6gal1^−/−^* rats and UC models in *St6gal1^+/+^* and *St6gal1^−/−^* rats. The colitis symptoms could be alleviated by *St6gal1* ablation, resulting in decreased proliferation and activation of CD4^+^ T‐cells cells. Conversely, the transfer of CD4^+^ T cells from *St6gal1^+/+^* UC rats aggravated the symptoms of colon inflammation. Attenuated signal transduction via TCR in *St6gal1^−/−^* CD4^+^ T cells accounts for low T‐cell proliferation. The number of CD4^+^ T cells in the colon tissues and peripheral blood of patients with UC is increased. The number of CD4^+^ T cells is increased in UC tissues.^[^ ^20b^ ^]^ The ablation of *ST6GAL1* promotes growth arrest in CD4^+^ T cells by at least two mechanisms: early by limiting TCR translocation to the lipid raft and consequent low T cell activation, and later by reducing the interaction of CD4^+^ T cells with antigen‐presenting cells. LPS has also been reported to be associated with UC.^[^ ^31^ ^]^ LPS upregulated *ST6GAL1* gene expression. An increase in pro‐inflammatory cytokines and a decrease in anti‐inflammatory cytokines often lead to UC.^[^ ^18^ ^]^ In addition, the levels of pro‐inflammatory cytokines were reduced, and the levels of anti‐inflammatory cytokines were increased in *St6gal1^−/−^* rats. Conversely, in patients with UC, proinflammatory cytokine levels are increased, and the levels of anti‐inflammatory cytokines are decreased. Th1, Th2, Treg, and Th17 cells are generated after the differentiation of naïve CD4^+^ T cells, and the balance between Th1/Th2 and Treg/Th17 cells maintains intestinal homeostasis by creating a balance between proinflammatory and anti‐inflammatory cytokines.^[^ ^32^ ^]^ The proportion of Treg/Th17 cells increased in *St6gal1^−/−^* rats. ST6GAL1 knockout induced the polarization of CD4^+^ T cells toward Treg cells and inhibited their polarization toward Th17 cells. Glycosylation plays a crucial role in Treg cell biology. Inhibiting α1,2‐mannosidase (preventing *N*‐glycan processing) impairs Treg migration to the lymph nodes.^[^ ^33^ ^]^ Additionally, the removal of *N*‐glycans results in an apparent loss of Treg cell capacity.^[^ ^34^ ^]^ Overexpression of NF‐κB often promotes the production of proinflammatory cytokines, which has important implications for UC development.^[^ ^24^ ^]^ *ST6GAL1* gene transcription is regulated by multiple promoters and is altered by glucocorticoids and cytokines.^[^ ^35^ ^]^ Therefore, we explored the correlation between ST6GAL1 and NF‐κB and discovered that ST6GAL1 expression is positively correlated with NF‐κB levels. The transcription factor *NF‐κB* binds to the promoter of *ST6GAL1* and promotes its transcription. High expression of ST6GAL1 in patients with UC increases NF‐κB production and induces the gradual accumulation of ST6GAL1. This may explain why patients with UC often experience repeated and aggravated symptoms during clinical treatment. Blockade of B cell‐activating factors attenuates DSS‐induced chronic colitis by inhibiting NF‐κB activation.^[^ ^36^ ^]^ Similarly, gut relief attenuates dextran sulfate sodium‐induced colitis by inhibiting NF‐κB signaling in mice.^[^ ^37^ ^]^

T‐cell activation is accompanied by the differential expression of glycosyltransferases, glycosidases, and their substrates, and these differences play direct and powerful roles in regulating the T‐cell response. *ST6GAL1* deficiency inhibits the α2,6‐sialylation of TCR, suppresses the entry of TCRs into lipid rafts on CD4^+^ T‐cells, attenuates TCR signaling strength, further downregulates the expression of NF‐κB, and reduces the production of proinflammatory cytokines to relieve UC. *ST6GAL1* can regulate the biological function of multiple glycoproteins; therefore, widespread changes in α2,6‐sialylated proteins make it difficult to determine the role of ST6GAL1 in T cell activation by individual glycosylated proteins. However, our observations revealed that UC pathogenesis is partially responsible for the functional alterations associated with the hyperexpression of ST6GAL1 in CD4^+^ T cells, which is a potential novel target for the clinical treatment of UC.

## 4 Experimental Section

### Generation of a St6gal1 Knockout Rat

The *St6gal1^−/−^* rat model was generated on a Sprague‐Dawley background using CRISPR/Cas9 technology. The *St6gal1* rat gene, located on chromosome 11q23, comprises six exons. ATG initiation codons were in exon 2, and TGA stop codons were in exon 6. Exons 2, 3, 4, 5, and 6, the most upstream exons among the possible exons, were targeted to disrupt the expression of all isoforms. Aiming at the *ST6GAL1* gene, four gRNAs were designed and synthesized: 3358‐ST6GAL1‐5S1, 3358‐ST6GAL1‐3S1, 3358‐ST6GAL1‐5S2, and 3358‐ST6GAL1‐3S2 (Table S1, Supporting Information). The sgRNA and Cas9 protein complexes (Integrated DNA Technologies) were microinjected into the cytoplasm of fertilized pronuclear rat eggs, and the surviving eggs were transferred to the uteri of pseudo‐pregnant recipient female rats. Genotyping was performed using DNA extracted from the tails of 2‐week‐old F0 rats. The genotypes of the newborn rats were identified by PCR and sequencing, and three *St6gal1* heterozygous (*St6gal1* ^+/−^) F0 generation rats were obtained (Table S1, Supporting Information). Rats heterozygous for these mutations were outcrossed with *St6gal1* homozygous wild‐type (*St6gal1^+/+^*) rats, and 22 (6 *St6gal1^+/−^* and 16 *St6gal1^+/+^*) F1 generation rats were obtained (Table S1, Supporting Information). Offspring of *St6gal1^+/−^* matings were used in all experiments.

### Genotyping

*St6gal1^+/+^* and *St6gal1^−/−^* rats were obtained by crossing heterozygous *St6gal1^+/−^* rats. Genotypes were evaluated using PCR. PCR conditions were as follows: 94 °C for 5 min, followed by 30 cycles of 94 °C for 30 s, 58.8 °C for 30 s, and 72 °C for 30 s. The primers used are listed in Table S2, Supporting Information. All animal experiments were approved by the Ethics Committee of Dalian Medical University (No. AEE17013).

### Collection of Human Tissue

Tissue and peripheral blood samples were collected from 73 HCs and 74 patients with UC recruited from the Department of Gastroenterology of Dalian University Affiliated Xinhua Hospital (Table S3, Supporting Information). UC diagnosis was based on clinical symptoms and endoscopic and histological findings. This study was approved by the Ethics Committee of Dalian University‐affiliated Xinhua Hospital (2022‐036‐01). All healthy volunteers and patients with UC completed informed consent before sample collection.

### Cells and Culture Conditions

The Jurkat cell line (ATCC Number: TIB‐152) was obtained from the American Type Culture Collection (ATCC, USA) and authenticated by STR profiling. Jurkat cells were maintained in Roswell Park Memorial Institute 1640 medium (Gibco, USA) with 10% Fetal Bovine Serum (Gibco, USA), penicillin (60 µg mL^−1^), and streptomycin (100 µg mL^−1^) (NCM Biotech, China) in 5% CO_2_ at 37 °C.

### Establishment of ST6GAL1 Gene Knockdown and ST6GAL1‐Restored Cell Lines

The pLKO.1 shRNA lentivirus system was used to generate a lentivirus encoding shRNA against human *ST6GAL1* (s*hST6GAL1*). *shST6GAL1* (sense: TAATACGACTCACTATAGGG, antisense: CTGGAATAGCTCAGAGGC, vector: psi‐LVRU6GP) and negative control (shall) were purchased from GeneCopoeia (China). ST6GAL1‐knockdown Jurkat cells were obtained using puromycin (1.5 µg mL^−1^).

To prepare *ST6GAL1* reintroduced cells, pEZ‐M77‐ST6GAL1 mutant expression vectors resistant to siRNAs expressed in shST6GAL1 cells were prepared. The pEZ‐M77‐ST6GAL1 vector was transfected into *ST6GAL1^−/^* ^−^ cells using Lipofectamine, and *ST6GAL1*‐restored Jurkat (*shST6GAL1‐RE*) and CD4^+^ T‐cells (*ST6GAL1^RE^*) cells were obtained after selection with 300 µg mL^−1^ neomycin.

### Tissue Immunofluorescence

Colon tissue sections from humans and rats were dewaxed in xylene, hydrated in ethanol, and boiled in sodium citrate buffer to expose the antigens. Then, 3% H_2_O_2_ was used to block endogenous peroxidase activity, and 3% bovine serum albumin (BSA) was used for blocking. Tissue sections were incubated overnight at 4 °C with rabbit monoclonal anti‐CD4 (1:300; Abcam) and biotin‐labeled Sambucus nigra agglutinin (SNA, 1:500; Vector). Sections were incubated with Alexa Fluor 594‐conjugated goat anti‐rabbit IgG (1:1000, Beyotime, China) or Alexa Fluor 488‐conjugated streptavidin (1:1000, Beyotime, China) for 1 h at 37 °C. Finally, the blocked slices were observed and imaged under a fluorescence microscope (Leica, Germany), and the percentage of positive areas were calculated using ImageJ software (National Institute of Mental Health, USA).

### Flow Cytometry

Rat spleens and Peyer's patches were ground, and the cells were collected by centrifugation at 1200 rpm for 3 min. Cells were incubated with Leukocyte Activation Cocktail, with BD GolgiPlug (BD Biosciences) at 37 °C for 2 h for stimulation, then were incubated with 3% BSA to block Fc receptors, and then stained with FITC‐anti‐CD4, APC‐anti‐CD8, PE‐anti‐CD25, and PE‐anti‐CD69 (1:50, eBioscience) antibodies for 30 min at 25 °C. For FoxP3 staining, the membranes were lysed using a FoxP3/transcription factor fixation/permeabilization kit (Thermo Fisher Scientific). Subsequently, the cells were stained with PE‐anti‐FoxP3 (1:50, eBioscience) and PE‐anti‐IL‐17 (1:50, eBioscience) antibodies for 30 min at room temperature. Finally, the cells were detected using a BD C6 Plus instrument (Becton, Dickinson and Company, USA) and analyzed using the FlowJo‐V10 software (Flow Cytometry Software Inc., USA). Human peripheral blood was used directly with erythrocyte lysates using the same procedure.

### Dual‐Luciferase Reporter Gene Assay

Jurkat cells were cultured in 96‐well plates at a density of 3.0 × 10^4^ cells well^−1^. Lipofectamine 3000 (Invitrogen, USA) was used to co‐transfect Jurkat cells with 0.2 ng of pGL3‐ST6GAL1‐WT or pGL3‐ST6GAL1‐MUT (Wuhan GeneCreate Biological Engineering Co., Ltd.) and 2 ng of pRL‐TK (Promega, Madison, Wisconsin) with pcDNA3.1‐NF‐κB1. After 48 h of transfection, the relative luciferase activity was calculated by normalizing firefly luciferase to Renilla luciferase.

### Statistical Analysis

The data were analyzed using a non‐parametric *t*‐test, Tukey's test, one‐way analysis of variance (ANOVA), or Spearman correlation analysis using GraphPad Software (version 9.0; GraphPad Inc., La Jolla, CA, USA). All data are presented as mean ± standard error of the mean (*n* ± 3), and differences with a *p*‐value < 0.05 were considered statistically significant. *p < 0.05, **p < 0.01, ***p <  0.001, ****p < 0.0001.

All other methods are included in the Supporting Information.

## Conflict of Interest

The authors declare no conflict of interest.

## Author Contributions

W.L. conceived the project and designed the research. Q.F. and W.L. wrote the paper. Q.F., Me.L., W.Z., K.Z., and Mi.L. performed the experiments and analyzed the experimental data. All the authors reviewed the results and approved the final version of the manuscript.

## Supporting information

Supporting Information

## Data Availability

The data that support the findings of this study are available from the corresponding author upon reasonable request.
