# Supplementary material for: Hyper α2,6‐Sialylation Promotes CD4+ T‐Cell Activation and Induces the Occurrence of Ulcerative Colitis
Source: Adv Sci (Weinh). 2023 Jul 9;10(26):2302607. doi: 10.1002/advs.202302607 (PMC10502867; doi:10.1002/advs.202302607)
Supplement: Supplementary file 1 — Supporting Information [file ADVS-10-2302607-s001.pdf]

ADVANCED SCIENCE
Open Access

Supporting Information

for *Adv. Sci.*, DOI 10.1002/advs.202302607

Hyper α2,6-Sialylation Promotes CD4$^+$ T-Cell Activation and Induces the Occurrence of Ulcerative Colitis

*Qingjie Fan, Mechou Li, Weiwei Zhao, Kaixin Zhang, Ming Li and Wenzhe Li**

Supporting Information

Hyper α2,6-sialylation promotes $CD4^+$ T cell activation and induces the occurrence of ulcerative colitis

*Qingjie Fan [1, 2], Mechou Li [2], Weiwei Zhao [2], Kaixin Zhang [2], Ming Li [2], Wenzhe Li [1, *]*

*Corresponding author. Email: liwenzhe@stu.edu.cn

This word file includes:

Supplementary Materials and Methods

Supplementary Figure 1 to 12

Supplementary Table 1 to 4

Supplementary Materials and Methods

Bioinformatics analysis

Transcriptomic data

We selected collated microarray experiments (GSE179285) from the Gene Expression Omnibus (GEO) database browser (www.ncbi.nlm.nih.gov/sites/GDSbrowser/). The database contains a total of 31 healthy volunteers' and 23 UC patients' colon biopsies. Transcripts were analyzed via the online platform GEO2R (www.ncbi.nlm.nih.gov/geo/geo2r/) for analysis. Compared to healthy volunteers, gene expression data of UC patients are expressed as log_2 Fold change (Log_2 FC).

Single cell sequencing data

Human Antigen Receptor Consortium (huARdb, https://huarc.net/database) was used to analyze the composition of colonic immune cells in healthy controls and UC patients. The site uses visualization tools to show the number of various types of immune cells in the colon of healthy controls and UC patients, respectively.

Induction of ulcerative colitis

St6gal1$^{+/+}$ or *St6gal1*$^{-/-}$ rats were administered with a 5% DSS (w/v, Coolaber) in the drinking water for seven days, then the mice were given normal drinking water for 1 days, and rats were sacrificed on day eight. The control group was drinking normal water every day. During animal modeling, the body weight, fecal shape, and blood in stool of the four groups of *St6gal1*$^{+/+}$, St6gal1$^{-/-}$, *St6gal1*$^{+/+}$ + DSS and *St6gal1*$^{-/-}$ + DSS rats were recorded daily. The disease activity index (DAI) of UC-modeled rats was calculated based on their body weight, fecal shape, and blood in stool, as shown in Supplementary Table 4.

Hematoxylin/ Eosin (H&E) Staining and histopathological evaluation

Human and rat colon samples were cut into small sections, placed in 4% tissue cell fixative (Solabio) for 24 h, and embedded in paraffin wax. Wax blocks were sliced into 5 μm slices by a microtome (Leica). Sections were dewaxed in xylene and hydrated in ethanol and stained with hematoxylin-eosin using the H&E staining kit, as described in the instructions (Solabio). Colon sections were imaged by microscopic imaging system (Leica), and the histopathology scores according to specific criteria. The pathology score includes both cell infiltrate and architecture, and the sum of the scores is the total pathology. One parameter is cell infiltrate (normal, 0; mild in mucosa, 1; moderate in mucosa, 2; marked in mucosa, 3; moderate/severe in mucosa and submucosa, 4; transmural, 5), and anther is architecture (no erosion, 0; focal erosion, 1; focal ulceration, 2; extended ulcerations, 3).

Enzyme-linked immunosorbent assay (ELISA)

The serum was separated by centrifugation at 3000 rpm for 10 min, and the secretion of IL-2, IL-4, IL-6, IL-10, IL-13, IL-17 and IFN-γ cytokines was analyzed in HCs and UC patients using ELISA kits (ebioscience) according to the manufacturer's instructions.

RNA extraction and reverse transcription-real-time fluorescence quantitative PCR (RT-qPCR)

Total RNA was extracted from whole colon or cells using an ultrasonic fragmentation instrument (labCAN) and the RNeasy Mini Kit (Qiagen) according to the manufacturer's instructions. The RNA was then converted using the TransScript® Green One-Step qRT-PCR SuperMix kit (TransScript®) into cDNA, followed by quantitative real-time PCR according to the instructions provided by the manufacturer. The primers used are summarized in Supplementary Table 2. The colon and cell samples of human and rats were normalized to GAPDH expression levels and expressed as relative expression.

Rat CD4$^+$ T-cell isolation and activation

Rat spleens were ground in RPMI 1640, and the cell suspension was filtered using a 100-µm cell strainer. Erythrocyte lysate was added to the cell suspension and subsequently CD4$^+$ T-cells were isolated using cell separation magnets using anti-rat CD4 magnetic particles (ebioscience, USA) acting at 37°C for 30 min. CD4$^+$ T-cells were activated in the presence of anti-rat CD3 (5 µg/mL) and CD28 (3 µg/mL) under 5% CO_2 at 37°C.

CD4$^+$ T-cell adoptive transfer experiment

Firstly, 5% DSS was used to construct *St6gal1*$^{+/+}$ and *St6gal1*$^{-/-}$ UC rat models. Then, CD4$^+$ T-cells from spleen of rats were sorted and injected into *St6gal1*$^{-/-}$ rats by caudal vein, and 5% DSS was used to construct UC model. Untreated *St6gal1*$^{-/-}$ rats were used as controls.

CFSE assay

Cells were labeled with CFDA-SE (Beyotime, China) and seeded into six-well plates overnight. After 72 h, the cells were collected, washed with PBS, and resuspended in PBS. The fluorescence intensity of the cells was then measured on a BD Accuri™ C6 flow cytometer (BD Pharmingen, USA).[1]

Intestinal epithelial cell isolation and LPS stimulation

The intestinal epithelial cells were isolated from *St6gal1*$^{+/+}$ and *St6gal1*$^{-/-}$ male rats with the previous description [2]. Intestinal tissues were washed three times with PBS containing 2% penicillin/streptomycin. The tissue was cut into small pieces and resuspended in 15 mL of HBSS (40-50 mg tissue/mL) containing 400 U/mL DNase (Sigma-Aldrich), 500 U/mL collagenase type IV (Sigma-Aldrich) and 0.09 U/mL dispase II (Roche Applied System), and incubated for 60 min at 37ºC. The digest was washed thrice with cold PBS containing 2 mM EDTA, and filtered through a 70 µm nylon mesh. Finally, cells were resuspended in complete DMEM Advanced (Gibco, ThermoFisher Scientific) (containing 10% fetal bovine serum, 2 mM ultraglutamine (Lonza), 1% penicillin/streptomycin and 1% amphotericin B), and cultured at 37ºC and 5% CO_2. Adherent cells showed a fibroblast-like morphology and were passed to a new culture flask when cultures reached 90% confluence. Cells were stimulated with LPS (50 µg/mL) for 8 h in vitro, and detected the mRNA levels of *ST6GAL1* by qPCR.

CD4$^+$ T-cell polarization

Firstly, CD4$^+$ T-cells from rat spleen were sorted in vitro by magnetic bead sorting technique, and then cultured in vitro, TGF-β and other cytokines were added to the medium to induce CD4$^+$ T-cells to differentiate into Treg and Th17 cells as described previously.[3]

Lipid raft isolation

UltraRIPA Lipid Raft Extraction Kit (Funakoshi, Japan) was used to extract lipid raft proteins from cells. The kit includes Solution A and Solution B. Solution A allows extraction of cytosolic and non-lipid raft proteins, and then Solution B allows extraction of lipid raft proteins. The specific operations according to the instructions provided by the manufacturer.

Protein extraction, Western and lectin blot analyses

The cell suspension was centrifuged at 1,200 rpm for 3 min, and appropriate amounts of cell lysate, protease inhibitor and phosphatase inhibitor were added to the cell mass, followed by lysis on ice for 15-30 min to allow adequate lysis. After lysis, the supernatant was collected by centrifugation at 12,000 rpm for 10 min and the protein concentration of each sample was determined using the BCA Protein Concentration Assay Kit (Beyotime biotech, China).

Total proteins in the cell samples were disulfide bond breaking by β-mercaptoethanol, using sodium dodecyl sulfate-polyacrylamide gel electrophoresis (SDS-PAGE) to separate various proteins, and then transferred to nitrocellulose filter membranes (NC) (Merck Millipore, USA). Membranes were sealed with 5% BSA for 2 h at room temperature (RT), followed by overnight incubation at 4°C with anti-p-zap70, zap70, p-p38, p38, p-ERK, ERK, NF-KB, and GAPDH antibody (Cell Signaling Technology, MA) or Sambucus Nigra Lectin (SNA), Concanavalin A (ConA), Aleuria Aurantia Lectin (AAL), and Maackia Amurensis Lectin (MAL, Vector Laboratories, USA). Afterwards, the membranes were incubated with HRP-conjugated Streptavidin or HRP-conjugated Abs (Beyotime) for 1 h at RT and then the protein bands were visualized using Omni-ECL™Femto Light Chemiluminescence Kit (Shanghai Epizyme Bio-medical Technology Co., Ltd, China). Densitometry analysis was performed using ImageJ software (National Institutes of Health, USA).

Immunofluorescence

For cellular immunofluorescence, cells were fixed in 4% paraformaldehyde, and permeabilized with 0.1% Triton X-100. Subsequently, cells were blocked with 3% BSA at room temperature for 1 h. Subsequently, cells were incubated with rabbit monoclonal antibody Flotillin-1 (1:200, abcam, England) and p-p65(1:300, CST, USA) or mouse monoclonal antibody TCR (1:200, abcam, England) overnight at 4°C. Cells were incubated with Alexa Fluor 594-conjugated goat anti-rabbit IgG (1:1000, Beyotime, China) or goat anti-mouse Alexa Fluor 488 (1:1000, Beyotime, China) for 1 h at 37°C. Finally, cell suspensions were dropped on slides and cell staining was observed directly with a fluorescent microscope (Leica, Germany).

Electrospray ionization mass spectrometry

Mass spectrometry analysis was carried out using an LTQ-XL linear ion trap electrospray ionization mass spectrometer coupled with an HPLC system (Thermo Scientific, Bremen, Germany.), as described previously.[4]

RNA-seq experiments and analysis

RNA sequencing methods as described previously.[5]

Chromatin immunoprecipitation-real-time fluorescence quantitative PCR (CHIP-qPCR)

Chromatin immunoprecipitation was accomplished by using CHIP Assay Kit (Beyotime, China). First, cells were collected by centrifugation at 1200 rpm for 3 min, and 1% formaldehyde was added for cross-linking of proteins and DNA fragments. Subsequently, genomic DNA was disrupted by ultrasonic fragmentation. Anti-NF-κB (1:10, abcam, England) was added to the samples to precipitate NF-κB overnight at 4°C, and 50 μL of protein A+G agarose/Sal-mon sperm DNA was added the next day. Finally, the genomic DNA was extracted according to the TransScript® Green One-Step qRT-PCR SuperMix kit (TransScript®, China) instructions, and the resulting precipitates were analyzed by qPCR technique. The primer sequences used to detect the promoter region of the human ST6GAL1 gene were as shown in Supplementary Table 2.

Dual-luciferase reporter gene assay

The Jurkat cells were grown in a 96-well plate at the density of 3.0×10^4 cells per well. Then, Lipofectamine TM 3000 (Invitrogen, USA) was used to co-transfect Jurkat cells with 0.2 ng of pGL3-ST6GAL1-WT or pGL3-ST6GAL1-MUT (Wuhan GeneCreate Biological Engineering Co., Ltd.) and 2 ng of pRL-TK (Promega, Madison, Wisconsin) with a NF-κB1-pcDNA3.1. After transfection for 48 h, the relative activity of luciferase was calculated by normalizing firefly luciferase to renilla luciferase.

Reference

[1] K. Wang, Q. Gong, Y. Zhan, B. Chen, T. Yin, Y. Lu, Y. Zhang, H. Wang, J. Ke, B. Du, X. Liu, J. Xiao, *Front Cell Dev Biol* **2019**, *7*, 397, https://doi.org/10.3389/fcell.2019.00397.

[2] L. Hidalgo-Garcia, J. A. Molina-Tijeras, F. Huertas-Pena, A. J. Ruiz-Malagon, P. Diez-Echave, T. Vezza, M. J. Rodriguez-Sojo, R. Moron, P. Becerra-Massare, A. Rodriguez-Nogales, J. Galvez, M. E. Rodriguez-Cabezas, P. Anderson, *Acta Physiol (Oxf)* **2021**, *233* (2), e13699, https://doi.org/10.1111/apha.13699.

[3] S. Flaherty, J. M. Reynolds, *J Vis Exp* **2015**, (98), https://doi.org/10.3791/52739.

[4] C. Wang, Z. Wu, J. Yuan, B. Wang, P. Zhang, Y. Zhang, Z. Wang, L. Huang, *J Proteome Res* **2014**, *13* (2), 372, https://doi.org/10.1021/pr4010647.

[5] Q. Fan, Y. Wu, M. Li, F. An, L. Yao, M. Wang, X. Wang, J. Yuan, K. Jiang, W. Li, M. Li, *Cell Death Dis* **2021**, *12* (12), 1094, https://doi.org/10.1038/s41419-021-04388-y.

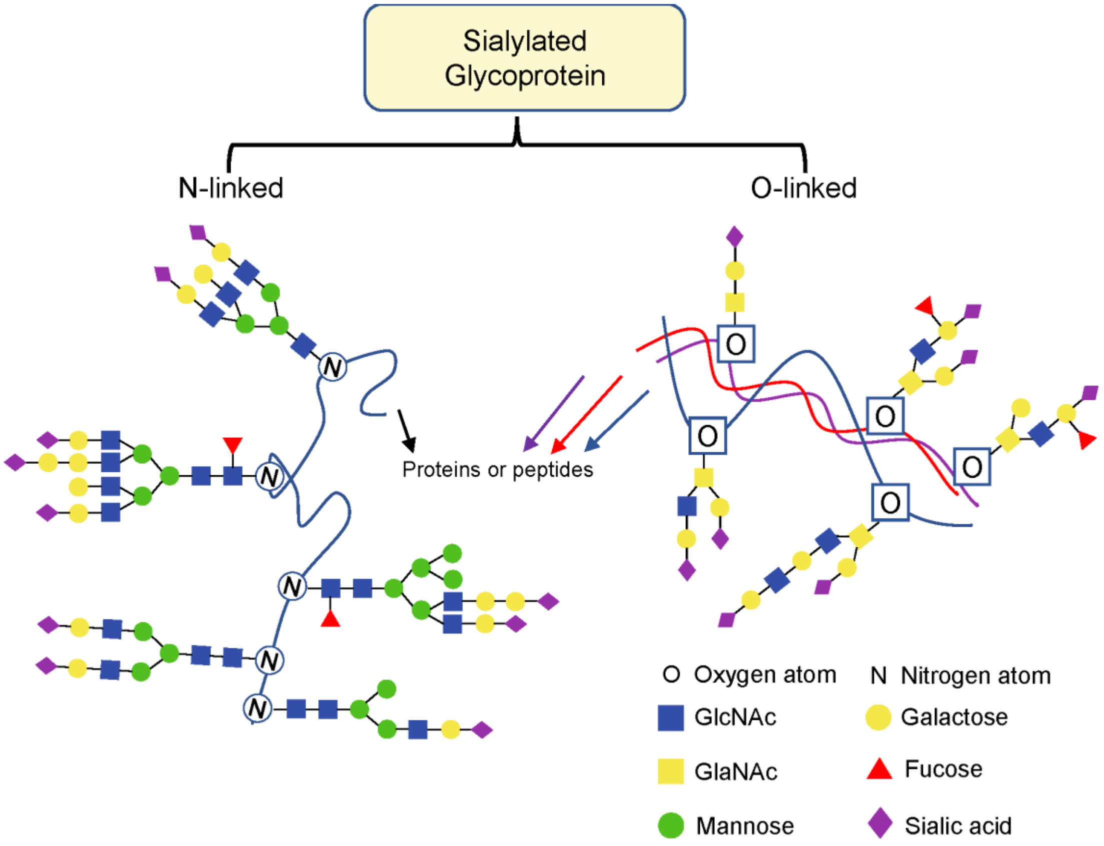

Gene name	Protein name	Function
ST6GAL1	Beta-galactoside alpha-2,6-sialyltransferase 1	Transfer NeuNAc from CMP-NeuNAc with an alpha-2,6-linkage to substrates
ST6GAL2	Beta-galactoside alpha-2,6-sialyltransferase 2	
ST6GALNAC1	Alpha-N-acetylgalactosaminide alpha-2,6-sialyltransferase 1	
ST6GALNAC2	Alpha-N-acetylgalactosaminide alpha-2,6-sialyltransferase 2	
ST6GALNAC3	Alpha-N-acetylgalactosaminide alpha-2,6-sialyltransferase 3	
ST6GALNAC4	Alpha-N-acetylgalactosaminide alpha-2,6-sialyltransferase 4	
ST6GALNAC5	Alpha-N-acetylgalactosaminide alpha-2,6-sialyltransferase 5	
ST6GALNAC6	Alpha-N-acetylgalactosaminide alpha-2,6-sialyltransferase 6	
ST3GAL1	Beta-galactoside alpha-2,6-sialyltransferase 1	Transfer NeuNAc from CMP-NeuNAc with an alpha-2,3-linkage to substrates
ST3GAL2	Beta-galactoside alpha-2,6-sialyltransferase 2	
ST3GAL3	Beta-galactoside alpha-2,6-sialyltransferase 3	
ST3GAL4	Beta-galactoside alpha-2,6-sialyltransferase 4	
ST3GAL5	Beta-galactoside alpha-2,6-sialyltransferase 5	
ST3GAL6	Beta-galactoside alpha-2,6-sialyltransferase 6	
ST8SIA1	Alpha-2,8-sialyltransferase 8A	Transfer NeuNAc from CMP-NeuNAc with an alpha-2,8-linkage to substrates
ST8SIA2	Alpha-2,8-sialyltransferase 8B	
ST8SIA3	Alpha-2,8-sialyltransferase 8C	
ST8SIA4	Alpha-2,8-sialyltransferase 8D	
ST8SIA5	Alpha-2,8-sialyltransferase 8E	
ST8SIA6	Alpha-2,8-sialyltransferase 8F	

Supplementary Figure 1. The diversity ofsialylated glycoproteins. Sialylated glycans can be attached to proteins or peptides through oxygen atom on serine/threonine ornitrogen atom on asparagine. Sialylated glycans can be linear or branched, comprised of multiple saccharides, including GlcNAc, GalNAc, mannose, fucose, galactose and so on.

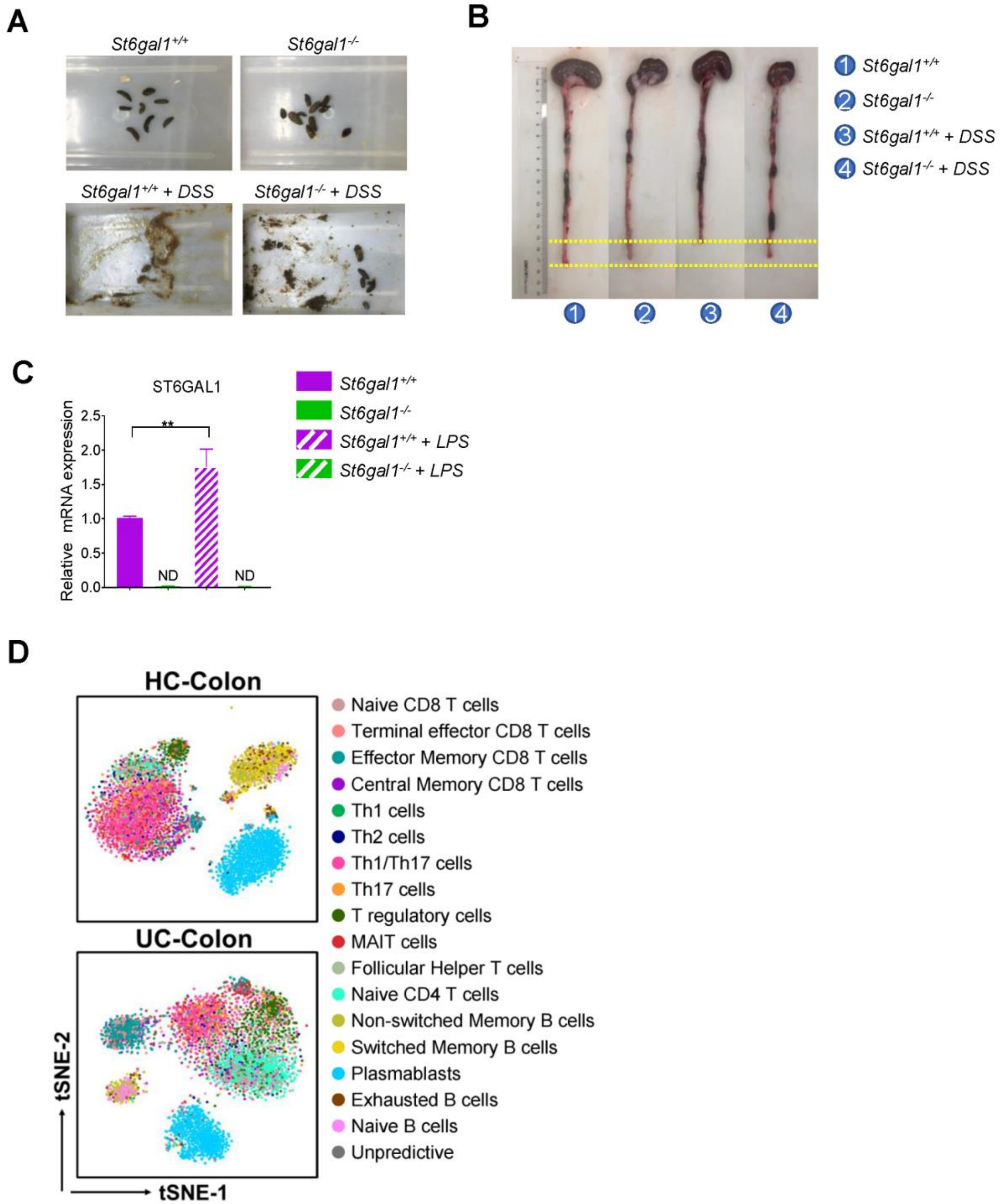

Supplementary Figure 2. The symptom of colitis and effect of LPS on *ST6GAL1* expression of intestinal epithelial cells in *St6gal*$^{+/+}$and *St6gal1*$^{-/-}$ male rats before and after UC modeling (n=10/group). *(A*) Shape of feces. *(B*) The colon length. (*C*) The effect of LPS on *ST6GAL1* expression of intestinal epithelial cells. (*D*) The tSNE plot from single-cell sequencing of colonic tissues from HCs and UC patients. $^{**}p < 0.01$.

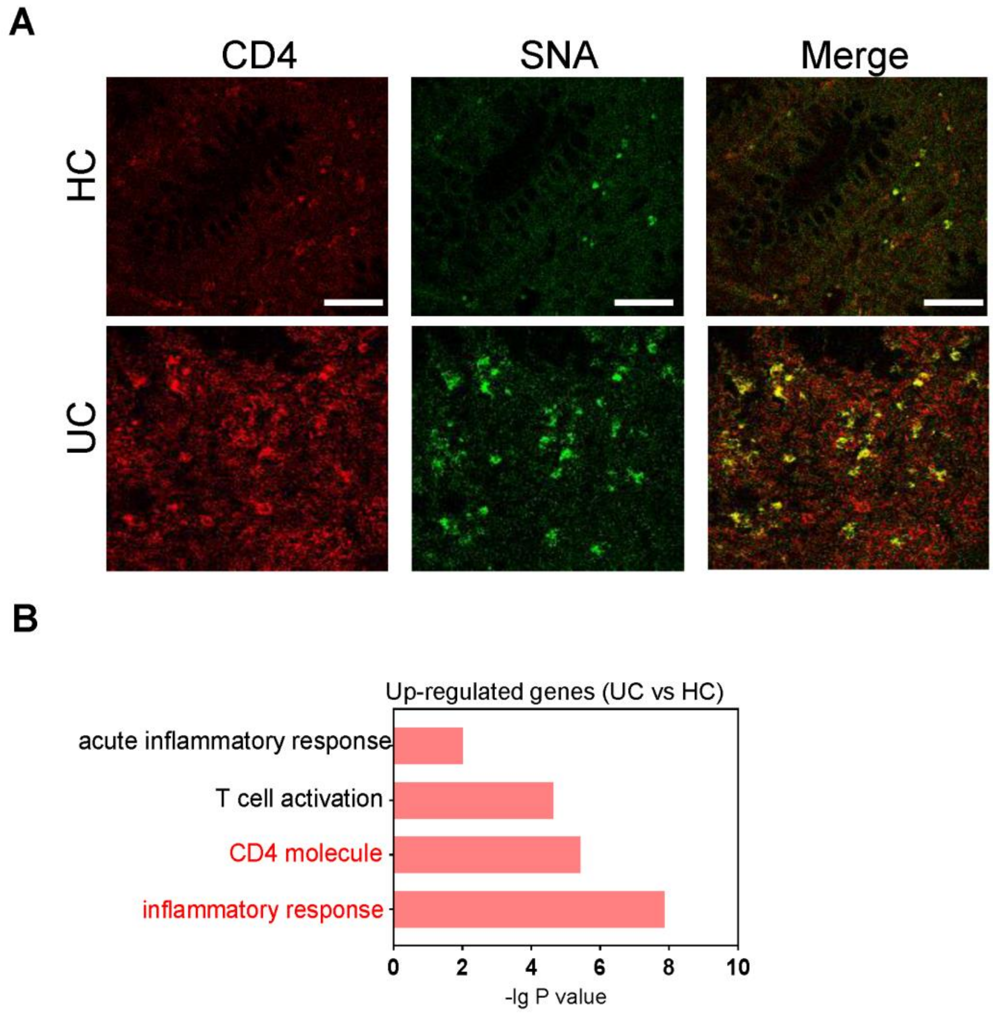

Supplementary Figure 3. Expression of CD4 in UC. *(A)* Expression of CD4 and its α2, 6-sialylation levels in HC and UC tissues. Scale bar, 100 µm. *(B)* GO enrichment analysis of $CD4^+$ T-cells and inflammatory responses in patients with UC.

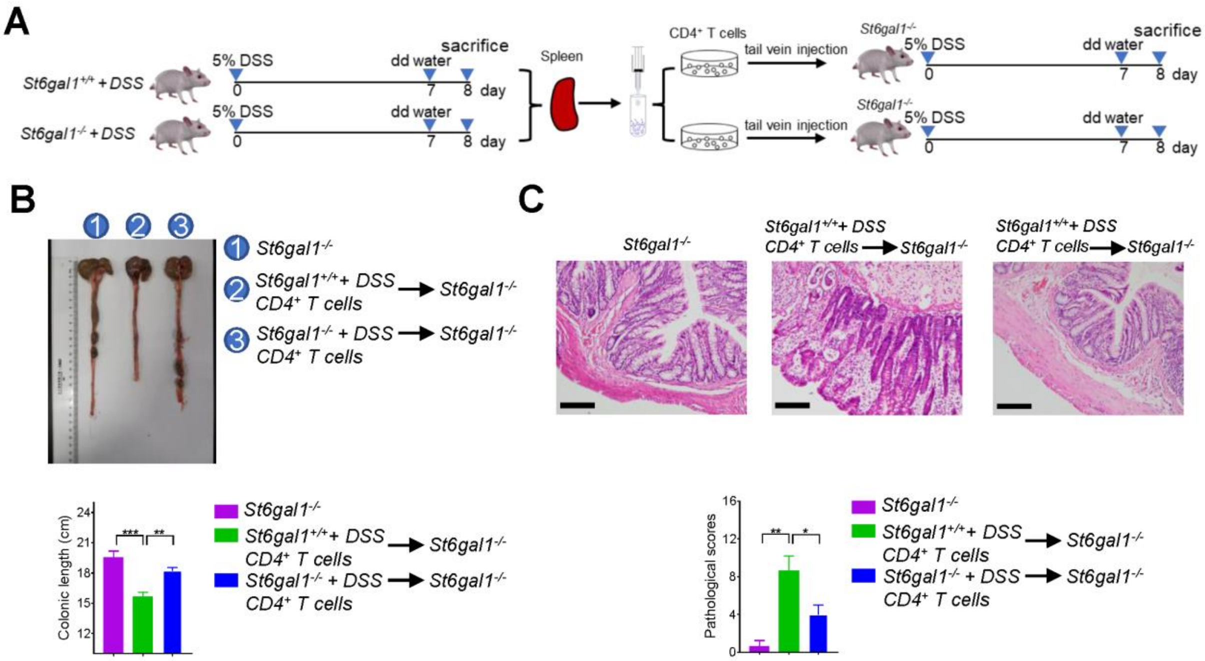

Supplementary Figure 4. CD4$^+$ T-cell adoptive transfer experiment. CD4$^+$ T-cells were isolated from *St6gal*$^{+/+}$and *St6gal1*$^{-/-}$ UC model rats (n=8/group). *St6gal1*$^{-/-}$ rats served as controls. *(A)* Overview of the CD4$^+$ T-cell adoptive transfer strategy. (*B*) Colon length. (*C*) HE and pathological score. Scale bar, 100 μm. *$p < 0.05$, **$p < 0.01$, ***$p < 0.001$.

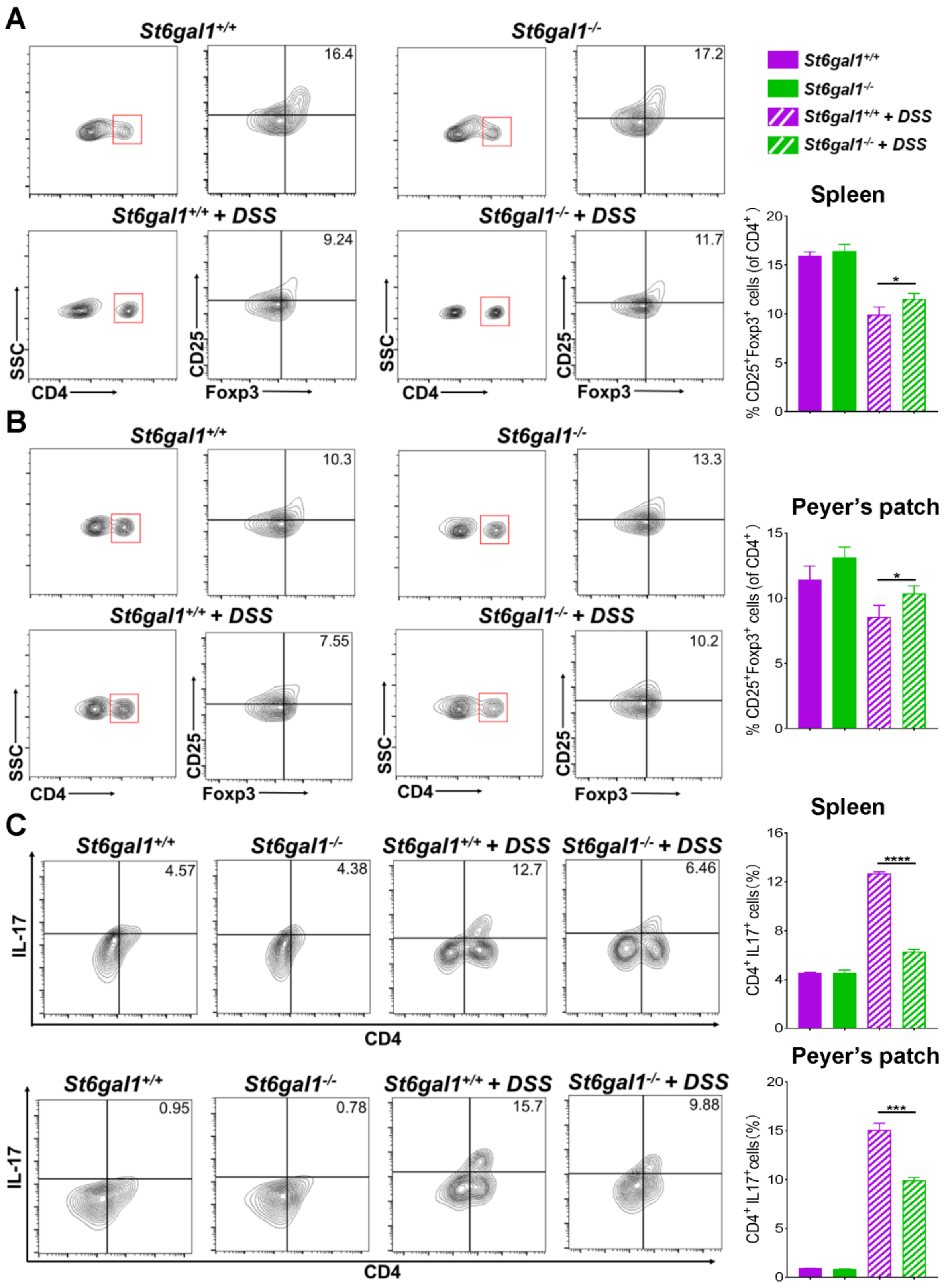

Supplementary Figure 5. Flow cytometry for Treg and Th17 cells. (*A*) Treg cells in rat spleen. Splenic cells were isolated from *St6gal*$^{+/+}$ and *St6gal1*$^{-/-}$ rats before and after UC

modeling. (*B*) Treg cells in rat Peyer's patches. Lymphocytes in Peyer's patches were isolated from *St6gal*$^{+/+}$ and *St6gal1*$^{-/-}$ rats before and after UC modeling. (*C*) Th17 cells in rat spleen and Peyer's patches. Lymphocytes were isolated from *St6gal*$^{+/+}$and *St6gal1*$^{-/-}$ rats before and after UC modeling. *$p < 0.05$, **$p < 0.01$, ***$p < 0.001$, ****$p < 0.0001$.

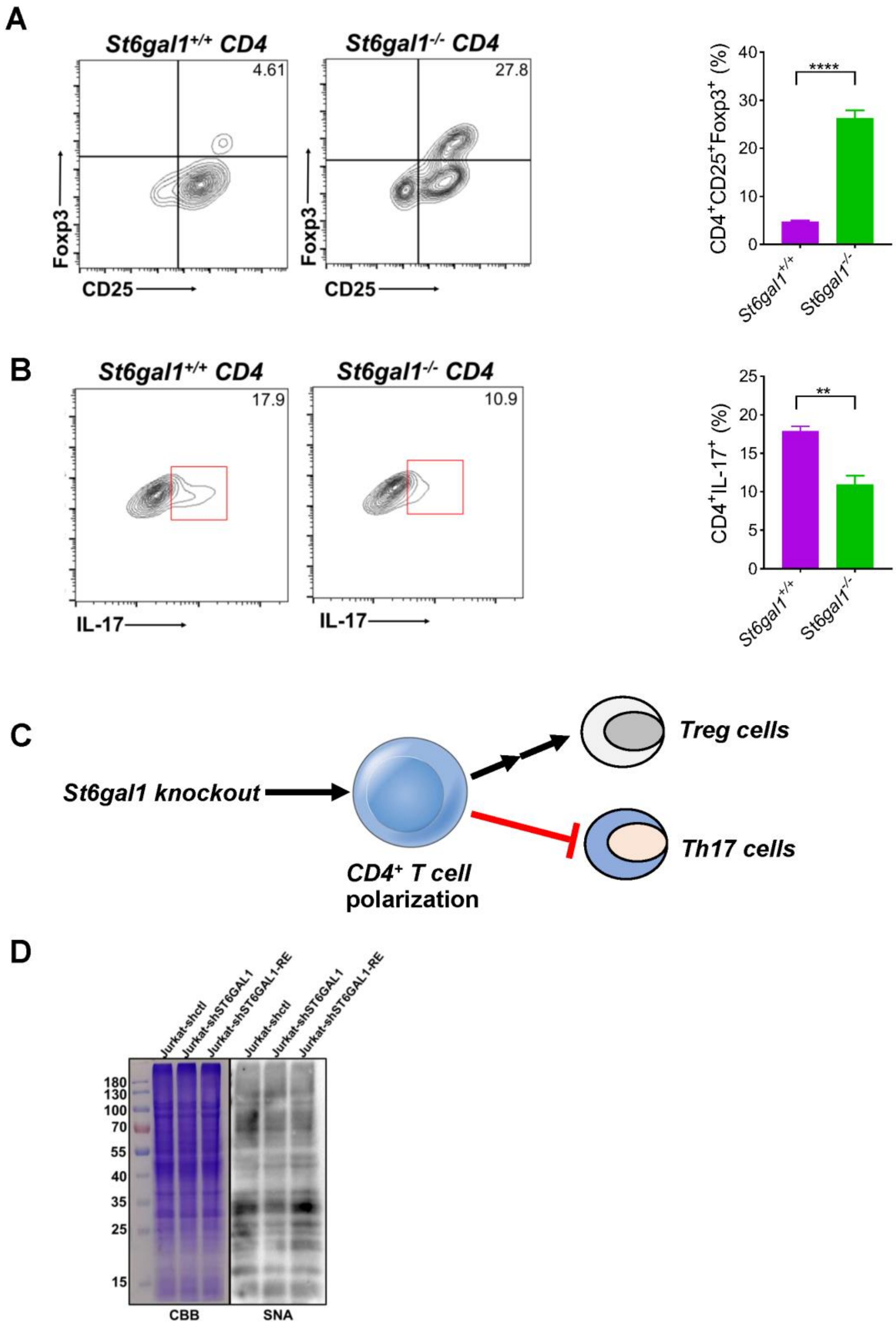

Supplementary Figure 6. CD4+ T-cell polarization. *(A*) Percentage of Treg cells. CD4+ T-cells were isolated from spleens of *St6gal*$^{+/+}$and *St6gal1*$^{-/-}$ rats, and CD4+CD25+Foxp3+ cells were analyzed. *(B*) Percentage of Th17 cells. CD4+ T-cells isolated from spleens of *St6gal*$^{+/+}$and *St6gal1*$^{-/-}$ rats, and the percentage of CD4+ IL17+ cells was determined. (*C*)

Schematic diagram of the effect of *ST6GAL1* on the polarization of $CD4^+$ T-cells. (*D*) Establishment of *ST6GAL1* gene knockdown Jurkat cells (*Jurkat-shST6GAL1* cells) and *ST6GAL1*-restored *Jurkat-shST6GAL1-RE* cells. The α2,6 sialylation level was detected by SNA blotting. Cell lysates were run on a 10% SDS-PAGE gel and immunostained with SNA. CBB was used as a loading control. $^{**}p < 0.01$, $^{****}p < 0.0001$.

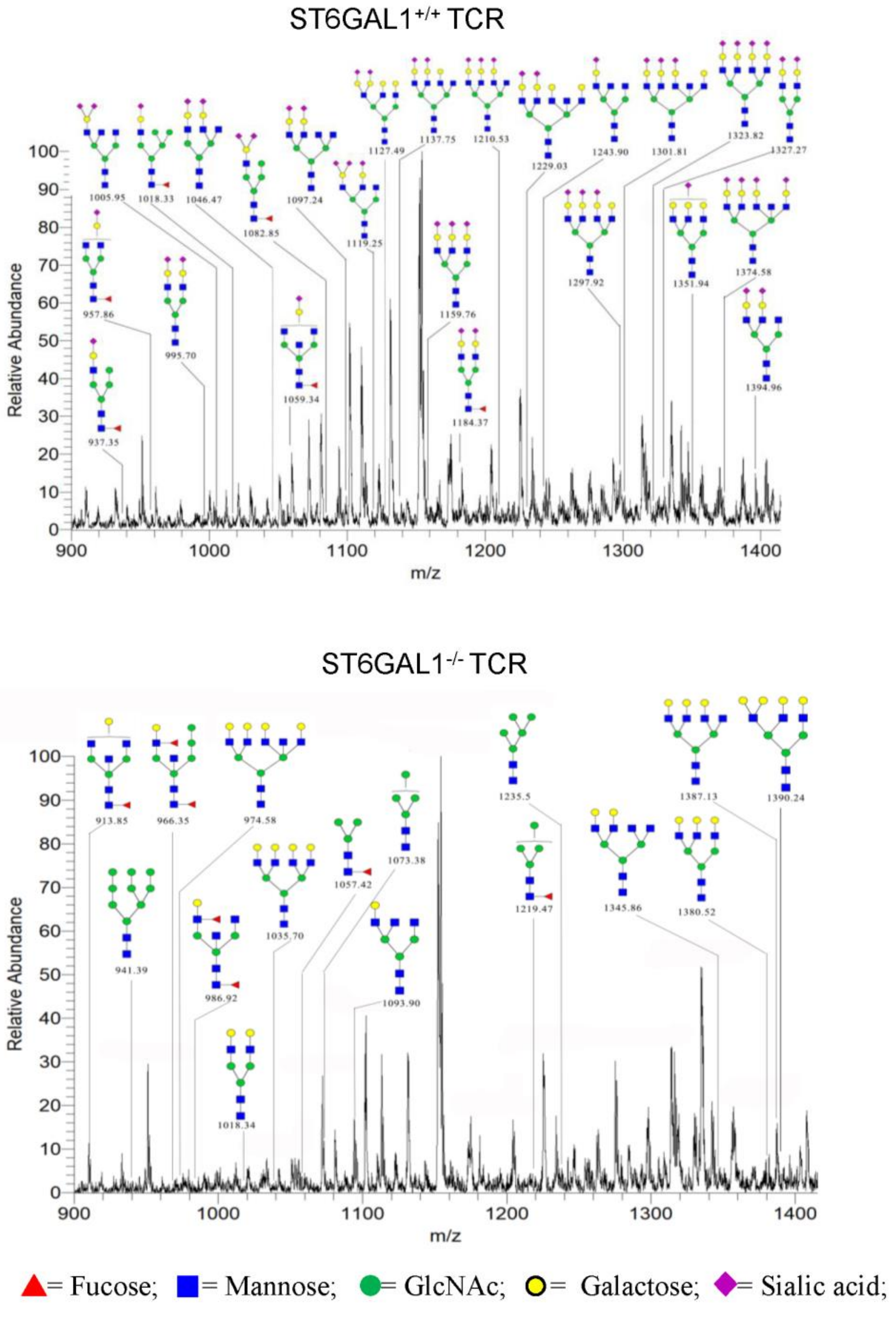

Supplementary Figure 7. N-glycan structures of the TCR on the surface of *St6gal1*$^{+/+}$ and *St6gal1*$^{-/-}$ CD4^{+} T-cells. Samples were loaded onto a Sep-Pak C18 SPE column, and the target N-glycans were eluted with 25% acetonitrile. The samples were injected via a Rheodyne loop with a 2 µl column and subsequently brought into the electrospray ion source by a stream of 50% methanol (v/v) at a flow rate of 200 µl/min.

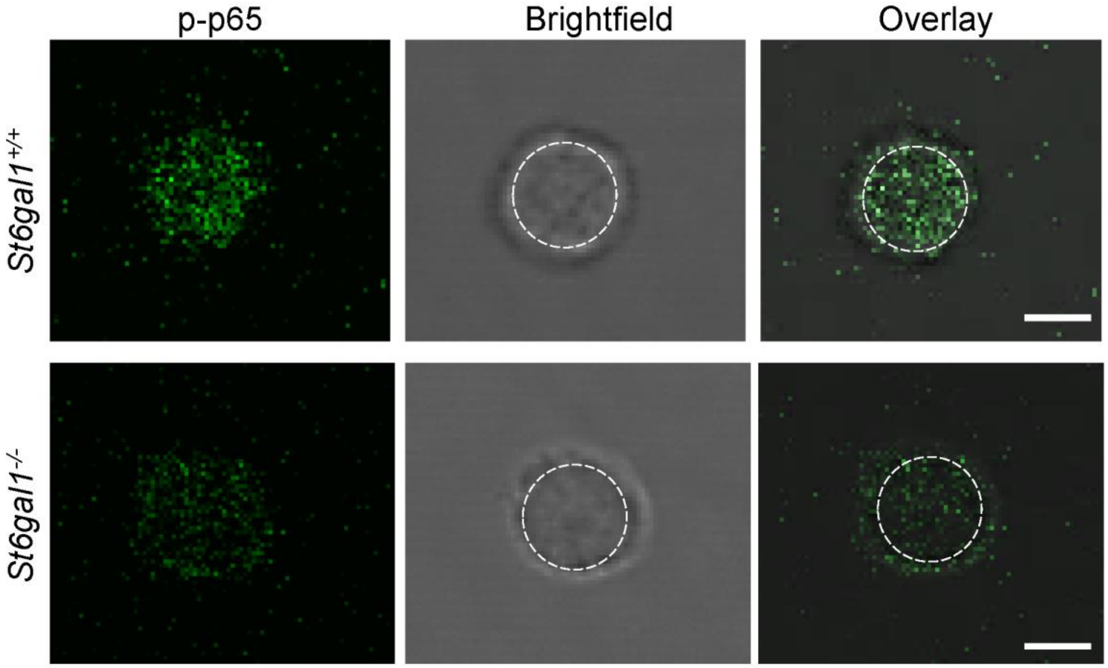

Supplementary Figure 8. Immunofluorescence staining. CD4^{+} T-cells from *St6gal1*$^{+/+}$ *and St6gal1*$^{-/-}$ spleen were probed with anti-p-NF-κB p65 (green) Ab after stimulation with anti-CD3/CD28 Abs for 15 min. Scale bar, 50 μm.

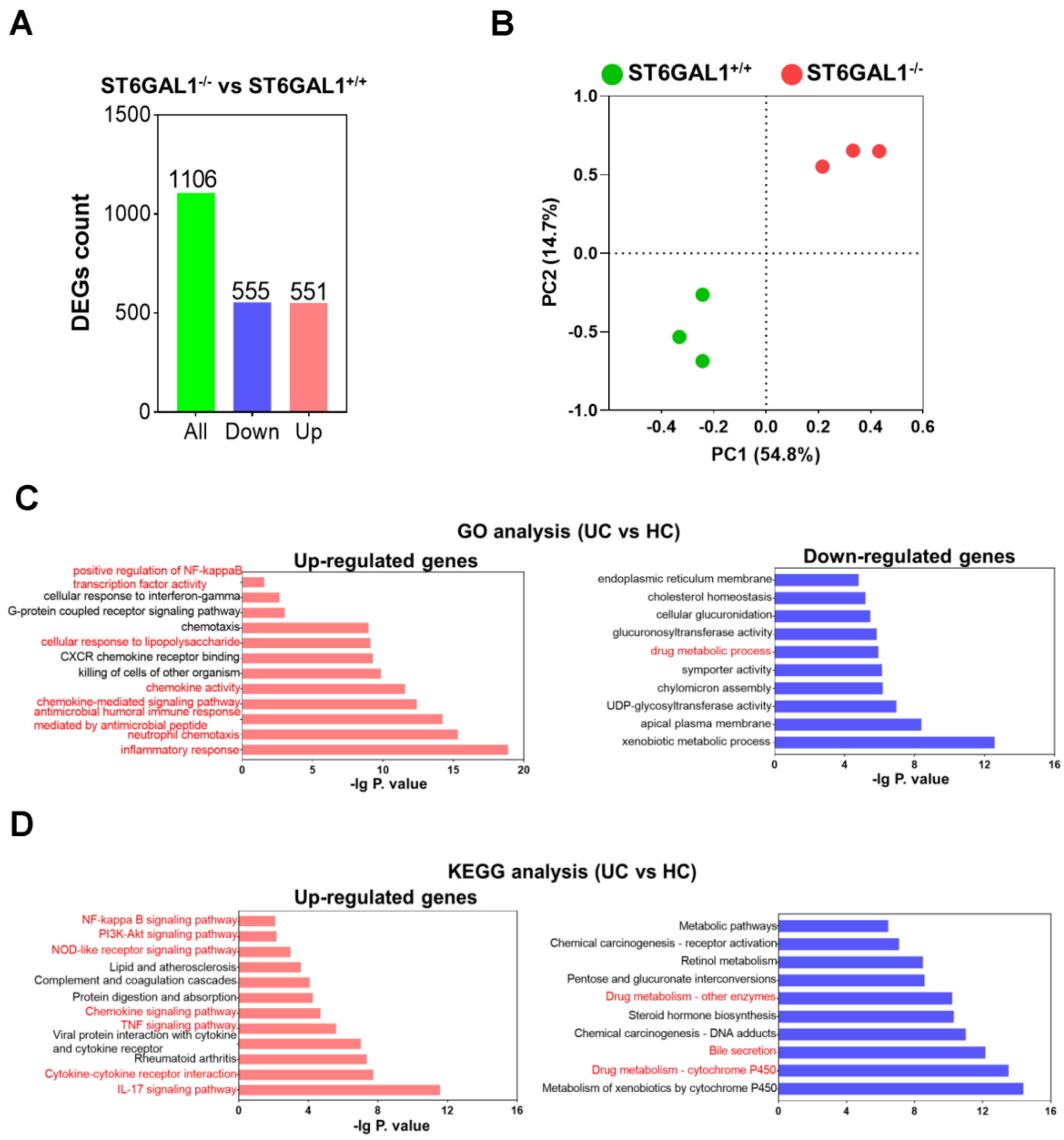

Supplementary Figure 9. Transcriptomics was used to analyze the effect of ST6GAL1$^{-/-}$ on CD4^{+} T-cells (n=3)**.** (*A*) Histogram of differentially-expressed genes in ST6GAL1$^{-/-}$ CD4^{+} T-cells compared with ST6GAL1$^{+/+}$ CD4^{+} T-cells. (*B*) PCA diagram of ST6GAL1$^{+/+}$ and ST6GAL1$^{-/-}$ groups. Bubble plots of GO and KEGG enrichment analysis of differentially-expressed genes in UC patients compared to HCs (GSE179285 database). (*C*) GO enrichment. (*D*) KEGG enrichment.

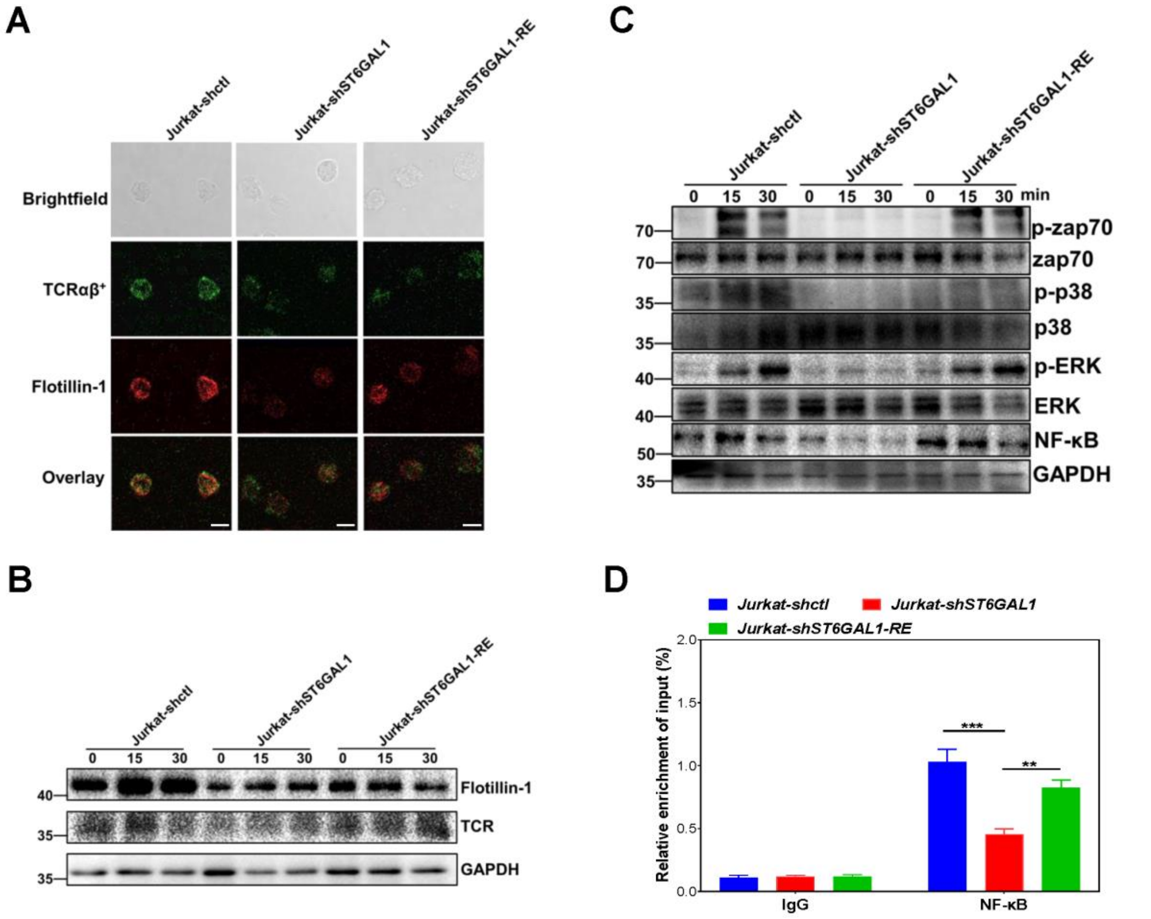

Supplementary Figure 10. Ablation of the *ST6GAL1* gene suppresses TCR translocation to lipid rafts and attenuates TCR signaling in Jurkat-cells. (*A*) Cellular immunofluorescence analysis the localization of TCR and Flotillin-1 in Jurkat-shctl, Jurkat-shST6GAL1 and Jurkat-shST6GAL1-RE cells. (*B*) Western blot. Cell lysates were run on 10% SDS-PAGE gel and immunostained with anti-TCR and anti-Flotillin-1 Abs. GAPDH was used as a loading control. (*C*) TCR signal pathway in Jurkat-shctl, Jurkat-shST6GAL1 and Jurkat-shST6GAL1-RE cells. Cells were lysed and cell lysates were run on 10% SDS-PAGE gel and immunostained. GAPDH was used as a loading control. (*D*) ChIP-qPCR. Binding of NF-κB to the promoter of the *ST6GAL1* gene was detected (n=3/group). Scale bar, 50 µm. $^{**}p < 0.01$, $^{***}p < 0.001$.

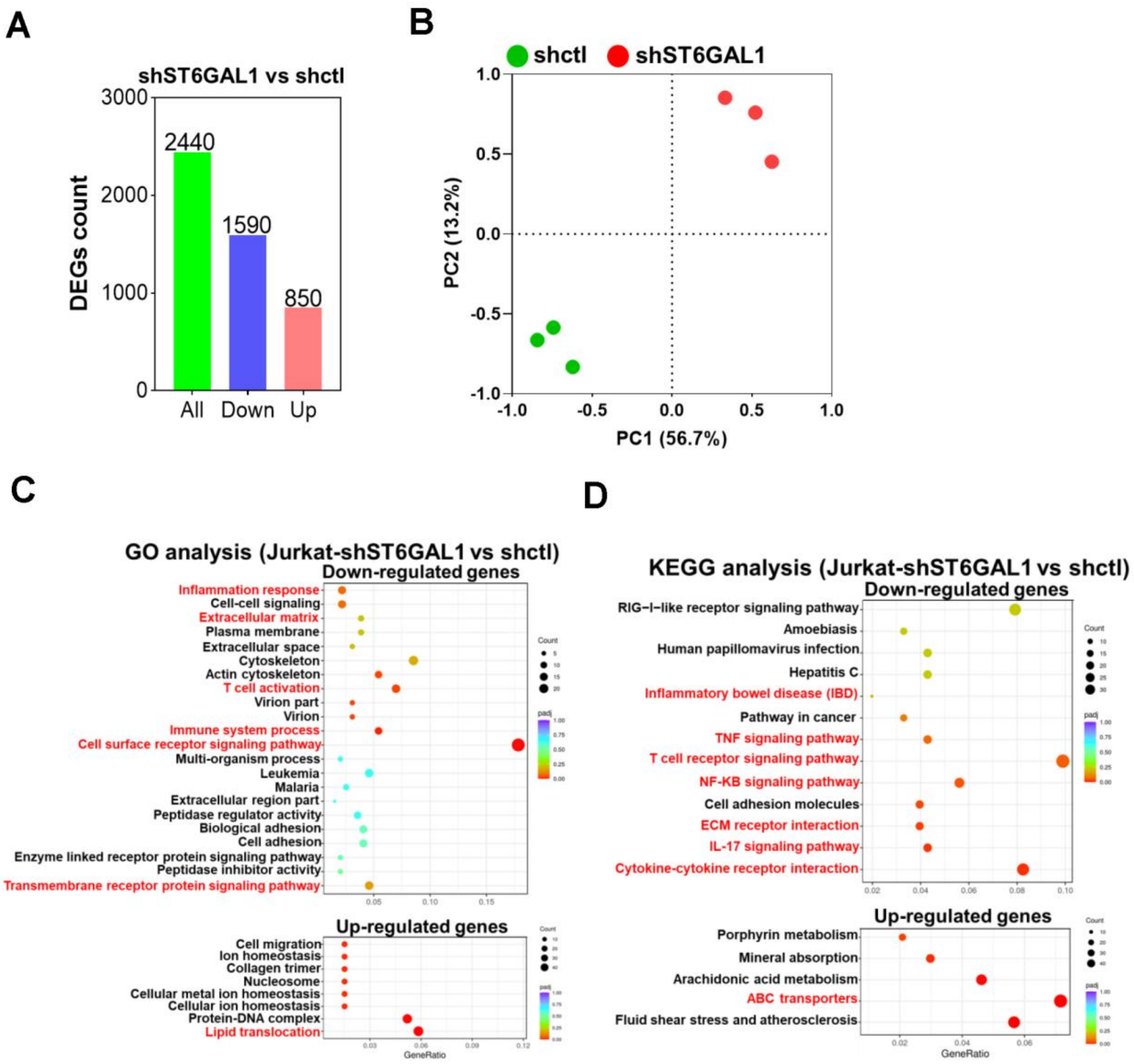

Supplementary Figure 11. Transcriptomics was used to analyze the effect of ST6GAL1 silencing on Jurkat cells (n=3). (A) Histogram of differentially-expressed genes in Jurkat-shST6GAL1 cells compared with Jurkat-shctl cells. (B) PCA diagram of Jurkat-shST6GAL1 and Jurkat-shctl groups. (C) Bubble plots of GO enrichment analysis of differentially-expressed genes in Jurkat-shST6GAL1 compared to Jurkat-shctl cells. (D) Bubble plots of KEGG enrichment analysis of differentially-expressed genes in Jurkat-shST6GAL1 compared to Jurkat-shctl cells.

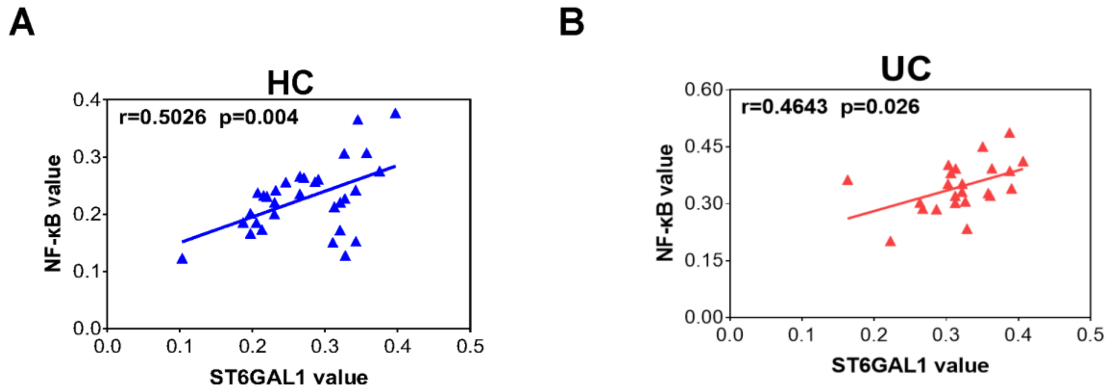

Supplementary Figure 12. Spearman correlation analysis. Correlation between colonic ST6GAL1 and NF-κB in HCs (*A*) and patients with UC (*B*) was analyzed using the GSE179285 database.

Supplementary Table 1. The CRISPR-Cas9 technique was used to construct ST6GAL1$^{-/-}$ rats

gRNA	gRNA sequence (5'→3')	PAM	ID	Gender	Color	Gene type	Generation
3358-ST6Gal-I-5S1	TCTGTGAGGGCTATGATCC	AGG	11	♀	white	+/-	F0
3358-ST6Gal-I-5S2	GCACTTTTAACTGATGT	AGG	12	♀	white	+/-	F0
3358-ST6Gal-I-3S1	TCACTTGTATCTAGCAA	GGG	17	♀	white	+/-	F0
3358-ST6Gal-I-3S2	CTCTTGCTCTGGAACTC	AGG	53	♂	white	-51144bp/+	F1
			60	♀	white	-51144bp/+	F1
			62	♀	white	-51144bp/+	F1
			64	♀	white	-51144bp/+	F1

Supplementary Table 2. Primer sequences for real-time RT-PCR

Gene name	Sense (5' → 3')	Antisense (5' → 3')
Human IL-2	CCC AAA CTC ACC AGG ATG CTC AC	GCA CTT CCT CCA GAG GTT TGA GTT C
Human IL-4	ACA GCA GTT CCA CAG GCA CAA G	CGT ACT CTG GTT GGC TTC CTT CAC
Human IL-6	GAC AGC CAC TCA CCT CTT CAG AAC	GCC TCT TTG CTG CTT TCA CAC ATG
Human IL-10	GGG TTG CCA AGC CTT GTC TGA G	CCT TGA TGT CTG GGT CTT GGT TCT C
Human IL-13	CAT GTA CTG TGC AGC CCT GGA ATC	GCA GAA TCC GCT CAG CAT CCT C
Human IL-17a	GAC CTC ATT GGT GTC ACT GCT ACT G	TCT TGT CCT CAG AAT TTG GGC ATC C
Human Foxp3	CAG AGA AGC AGC GGA CAC TCA ATG	CAG ACT CAG GTT GTG GCG GAT G
Human IFN-γ	CTG ACT TGA ATG TCC AAC GCA AAG C	CGA CCT CGA AAC AGC ATC TGA CTC
Human ST3GAL1	CCA CCC AGC CTT CAT CAA GTA TGT C	GAA GCC GTA CAA GTC CAC CTC ATC
Human ST3GAL2	TGG ACG GGC ACA ACT TCA TCA TG	GGC AGG TTC TTG GCA CTC TCA G
Human ST3GAL3	ATC CCT ACC CTT GGC AGT GTG G	CCA TGC GAA CGG TCT CAT AGT AGT G
Human ST3GAL4	AGT GAT AAG AAG CGG GTG CGA AAG	TTG GCA GGC TCA GCA GTT TGT C
Human ST3GAL5	GAG CAC AGG TAT AGC GTG GAC TTA C	GGA TCG TAC TTG GAC TCA GCT TCA C
Human ST3GAL6	GTG TGG TGG TTG GTA ATG GAG GAG	AGG TTG TCC TTC TCC CAA CTT CTT C
Human ST6GAL1	CCC CAA TCA GCC CTT TTA CAT CCT C	CCT GGT CAC ACA GCG TCA TCA TG
Human ST6GAL2	ACA GGC TGT ACT CCT CCA TGT CC	TGC TTG TTG GCG GTC AGG TAA TC
Human ST6GALNAC1	CTC TGT GAC CAG GTG AGT GCT TAT G	CGT GTA GCC GCT TCC AGA CTT C
Human ST6GALNAC2	TGA AGA ACT CCC TCG TCT CCT ACT G	GGC CGA TCT CAG CAT CAC ATA GTC
Human ST6GALNAC3	TTC CTG CTG GTT GTG CGT CTT G	GCC GCC TGT ATG TGT AGG AGA ATG
Human ST6GALNAC4	TGA GGA GAT CGT GGT CTA TGG GAT G	GTG CCA GGT ACA TCT GAC ACT CAT C
Human ST6GALNAC5	CTG GAC GGA TAC CTC GGA GTG G	ATT GTC ATT GTA AAC CAG CCA GTG C
Human ST6GALNAC6	CAA CCA CCA CCG CTT CAT CAC C	CTG CTT CTC CTC TGA CCC TCC TG
Human ST8SIA1	CCC AGC ATA ATT CGG CAA AGG TTT C	CCT CAA AGA TGG CTC TGT TCC TGT C
Human ST8SIA2	CTG CTC GTG GTC TTC CTC ATC TTC	GCT GGA TGT TGT GCT TGA TGC TTT C
Human ST8SIA3	GGG AGC CAG TGT GGA CAA GAA ATA G	GAT GCT GGG GTT GAA GGT GGT AAG

Human ST8SIA4	TCG AAA GGC TGG CTC TTC AAT CTT C	CTG CTC TTG ACC ACT GAC ACA TCT C
Human ST8SIA5	CAG CAT CAT CAC AGA GAG GTT CCA C	GTG TTG TAG AAG GCA GGC AGC AG
Human ST8SIA6	TGC TTC CTG CTG TGA TGC TGT TC	ACA AAA GGC TGG GAC ACT GGA AAC
Human NF-KB1	CAC TGT AAC TGC TGG ACC CAA GG	CGC CTC TGT CAT TCG TGC TTC C
Human GAPDH	GCA CCG TCA AGG CTG AGA AC	TGG TGA AGA CGC CAG TGG A
Rat IL-2	GCA GCG TGT GTT GGA TTT GAC TC	TGG CTC ATC ATC GAA TTG GCA CTC
Rat IL-4	CAA GGA ACA CCA CGG AGA ACG AG	TTC TTC AAG CAC GGA GGT ACA TCA C
Rat IL-6	ACT TCC AGC CAG TTG CCT TCT TG	TGG TCT GTT GTG GGT GGT ATC CTC
Rat IL-10	GGC AGT GGA GCA GGT GAA GAA TG	TGT CAC GTA GGC TTC TAT GCA GTT G
Rat IL-13	CTC GCT TGC CTT GGT GGT CTT G	TCT GGT CTT GTG TGA TGT TGC TCA G
Rat IL-17a	TGC CTG ATG CTG TTG CTG CTA C	GCG TTT GGA CAC ACT GAA CTT TGA G
Rat Foxp3	TCA CAC GCA TGT TCG CCT ACT TC	CTC ACT CTC CAC TCG CAC AAA GC
Rat IFN-γ	ACA ACC CAC AGA TCC AGC ACA AAG	CAC CGA CTC CTT TTC CGC TTC C
Rat ST6GAL1	GGT CTT TCT CCT GTT CGC AGT CAT C	TCT CCT GGC TCT TGG GCA TCT G
Rat GAPDH	GGC ACA GTC AAG GCT GAG AAT G	ATG GTG GTG AAG ACG CCA GTA

Supplementary Table 3. Patient characteristics

	HC	UC
Numbers of patients	73	74
Age (years)	42.2 ± 6.80	46.2 ± 3.12
Gender		
Male	37	38
Female	36	36
Disease duration (Months)		45.6 ± 12.24
Current therapy		
5-amiosalicylates		50
Immunosuppressants		0
Biologics		0
Nutritional therapy		24
Disease extent*		
E1		26
E2		24
E3		24

*According to the Montreal classification system.
HC, healthy controls; UC, ulcerative colitis.

Supplementary Table 4. Scoring of disease activity index (DAI)

Sores	Weight loss (%)	Stool consistency	Hemoccult
0	None	Normal	Normal
1	1-5		Hemoccult
2	6-10	Loose stools	Hemoccult+
3	11-15		Hemoccult++
4	16-20	Diarrhea	Rectal bleeding
